# “Mn-locking” effect by anionic coordination manipulation stabilizing Mn-rich phosphate cathodes†

**DOI:** 10.1039/d3sc03095d

**Published:** 2023-07-27

**Authors:** Wei Zhang, Yulun Wu, Yuhang Dai, Zhenming Xu, Liang He, Zheng Li, Shihao Li, Ruwei Chen, Xuan Gao, Wei Zong, Fei Guo, Jiexin Zhu, Haobo Dong, Jianwei Li, Chumei Ye, Simin Li, Feixiang Wu, Zhian Zhang, Guanjie He, Yanqing Lai, Ivan P. Parkin

**Affiliations:** a School of Metallurgy and Environment, Engineering Research Center of the Ministry of Education for Advanced Battery Materials, Hunan Provincial Key Laboratory of Nonferrous Value-Added Metallurgy, Central South University Changsha 410083 P. R. China zhangzhian@csu.edu.cn laiyanqing@csu.edu.cn; b Christopher Ingold Laboratory, Department of Chemistry, University College London London WC1H 0AJ UK i.p.parkin@ucl.ac.uk; c Electrochemical Innovation Lab (EIL), Department of Chemical Engineering, University College London London WC1E 7JE UK g.he@ucl.ac.uk; d Jiangsu Key Laboratory of Electrochemical Energy Storage Technologies, College of Materials Science and Technology, Nanjing University of Aeronautics and Astronautics Nanjing 210016 P. R. China xuzhenming@nuaa.edu.cn; e Department of Materials Science and Metallurgy, University of Cambridge Cambridge CB3 0FS UK

## Abstract

High-voltage cathodes with high power and stable cyclability are needed for high-performance sodium-ion batteries. However, the low kinetics and inferior capacity retention from structural instability impede the development of Mn-rich phosphate cathodes. Here, we propose light-weight fluorine (F) doping strategy to decrease the energy gap to 0.22 eV from 1.52 eV and trigger a “Mn-locking” effect—to strengthen the adjacent chemical bonding around Mn as confirmed by density functional theory calculations, which ensure the optimized Mn ligand framework, suppressed Mn dissolution, improved structural stability and enhanced electronic conductivity. The combination of *in situ* and *ex situ* techniques determine that the F dopant has no influence on the Na^+^ storage mechanisms. As a result, an outstanding rate performance up to 40C and an improved cycling stability (1000 cycles at 20C) are achieved. This work presents an effective and widely available light-weight anion doping strategy for high-performance polyanionic cathodes.

## Abstract 2

Our study introduced light-weight fluorine into Mn-rich cathode to trigger a “Mn-locking” effect: more robust Mn ligand framework, suppressed Mn dissolution, improved structural stability and enhanced electronic conductivity.

## Graphical abstract

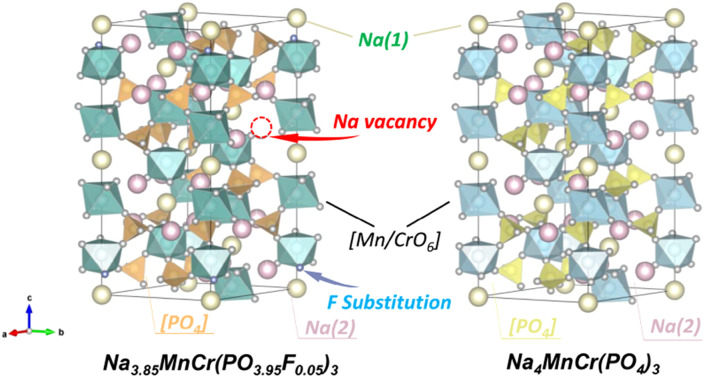

## Introduction

Nowadays, the development of energy storage systems (ESSs) is a critical project that needs to consider cost-effectiveness, high round-trip efficiency, environmental friendliness, and long lifespan, *etc.*^1–6^ The currently widely used lithium-ion batteries (LIBs) might be a candidate for this purpose.^7–10^ However, they will be excluded when the limited lithium resources and underlying political disputes are taken into consideration.^11–13^ Sodium-ion batteries (SIBs) emerge as promising alternatives due to the omnipresent availability of Na resources.^14–17^ Numerous efforts have been dedicated to developing high-performance cathodes for SIBs, which mainly include layered oxides and polyanionic compounds.^18–21^ Layered oxides (Na_*x*_MeO_2_, Me = Mg, Fe, Cu, Ni, Mn, *etc.*) are featured with facile synthesis and high capacities but are still confronted with inferior structural stability, noticeable volume change, and low operating voltage.^22–26^

In contrast, polyanionic compounds are endowed with excellent structural stability (strong covalent bond, *e.g.*, P–O bonds) and higher output voltage than oxides, which is associated with inductive effects of the polyanion groups (*e.g.*, (PO_4_)^3−^).^27–29^ Furthermore, the electrochemical potential tunability of polyanionic compounds is presumed to be a promising characteristic to increase overall voltage and activate multi-electron reactions (higher capacity) by involving different transition metals and adjusting the operating voltage window, which can compensate the lower output voltage of SIBs compared to LIBs due to the 0.3 V higher electrochemical potential of Na^+^/Na than that of Li^+^/Li.^12,18^ To illustrate, different transition metal redox couples in the same phosphate framework display different voltages *versus* Na^+^/Na, such as V^2+^/V^3+^ (∼1.6 V), Ti^3+^/Ti^4+^ (∼2.1 V), Fe^2+^/Fe^3+^ (∼2.5 V), V^3+^/V^4+^ (∼3.4 V), Mn^2+^/Mn^3+^ (∼3.6 V), V^4+^/V^5+^ (∼4.0 V) and Mn^3+^/Mn^4+^ (∼4.2 V) according to the reported works.^20,30–34^ It hence seems that manganese-rich (Mn-rich) phosphates can be good candidates as high-voltage cathodes.

Mn-rich phosphates cathodes chemistries hold long-lasting promise due to pressing requirements to reduce the dependence on those expensive resources (*e.g.*, Co, Ni, and V), increase the overall voltage, and extend life expectancy of batteries. For example, Na_4_MnCr(PO_4_)_3_ with reversible Mn^2+^/Mn^3+^ (3.6 V *versus* Na^+^/Na), Mn^3+^/Mn^4+^ (4.2 V) and Cr^4+^/Cr^3+^ (4.4 V) redox pairs was recently proposed,^35–37^ giving rise to the highest output voltage among phosphates cathodes. When accompanied by multi-electron reactions, Na_4_MnCr(PO_4_)_3_ could deliver an ultrahigh energy density of ∼566 W h kg^−1^ (1.4–4.6 V *versus* Na^+^/Na), which broke the record of the phosphates family and created great opportunities for the high-performance SIBs. Similar to other Mn-rich materials, nevertheless, it still suffers from the low intrinsic electronic conductivity and inferior cycling stability (obvious capacity fading within 20 cycles),^35^ which possibly stem from Mn^2+^ dissolution in the electrolyte and electrolyte decomposition at a high cut-off voltage (>4.5 V).^38–40^ It should be noted that the dissolution of Mn^2+^ is closely related to battery performance degradation by deteriorating the solid-electrolyte interphase (SEI) composition of anode surface and catalyzing the decomposition process of bulk electrolyte according to previous works, which has significantly impeded the practical application of almost all Mn-rich cathodes.^39,40^ To our knowledge, there is rare work to further modify Na_4_MnCr(PO_4_)_3_ and the capacity fading mechanism still remains elusive.

On the other hand, carbon coating and heteroatom doping can be efficient approaches to improve polyanionic materials but still face tremendous challenges. Carbon coating would inevitably increase the inactive mass of the electrode and thus lower the overall energy density. It is also challenging to form a uniform carbon coating layer and the conductive coating layer alone cannot solve all the above issues. The heteroatom doping can effectively tune the electronic structure and microstructures of cathode materials. As for the cation doping, heavy inactive metal ions (*e.g.*, Mo^6+^, Zr^4+^, Ca^2+^, and Cu^2+^, *etc.*) also lower the capacity. Therefore, it is highly desirable to develop a novel doping strategy based on a light-weight element with high electronegativity to increase the intrinsic electronic conductivity and stabilize the structure with a minimal capacity reduction. The highly electronegative fluorine can be a promising dopant candidate for its strong interaction with metals and relatively low atomic weight.^41–43^ The underlying mechanisms for doping enhancement of properties are still not well understood.

**Fig. 1:**
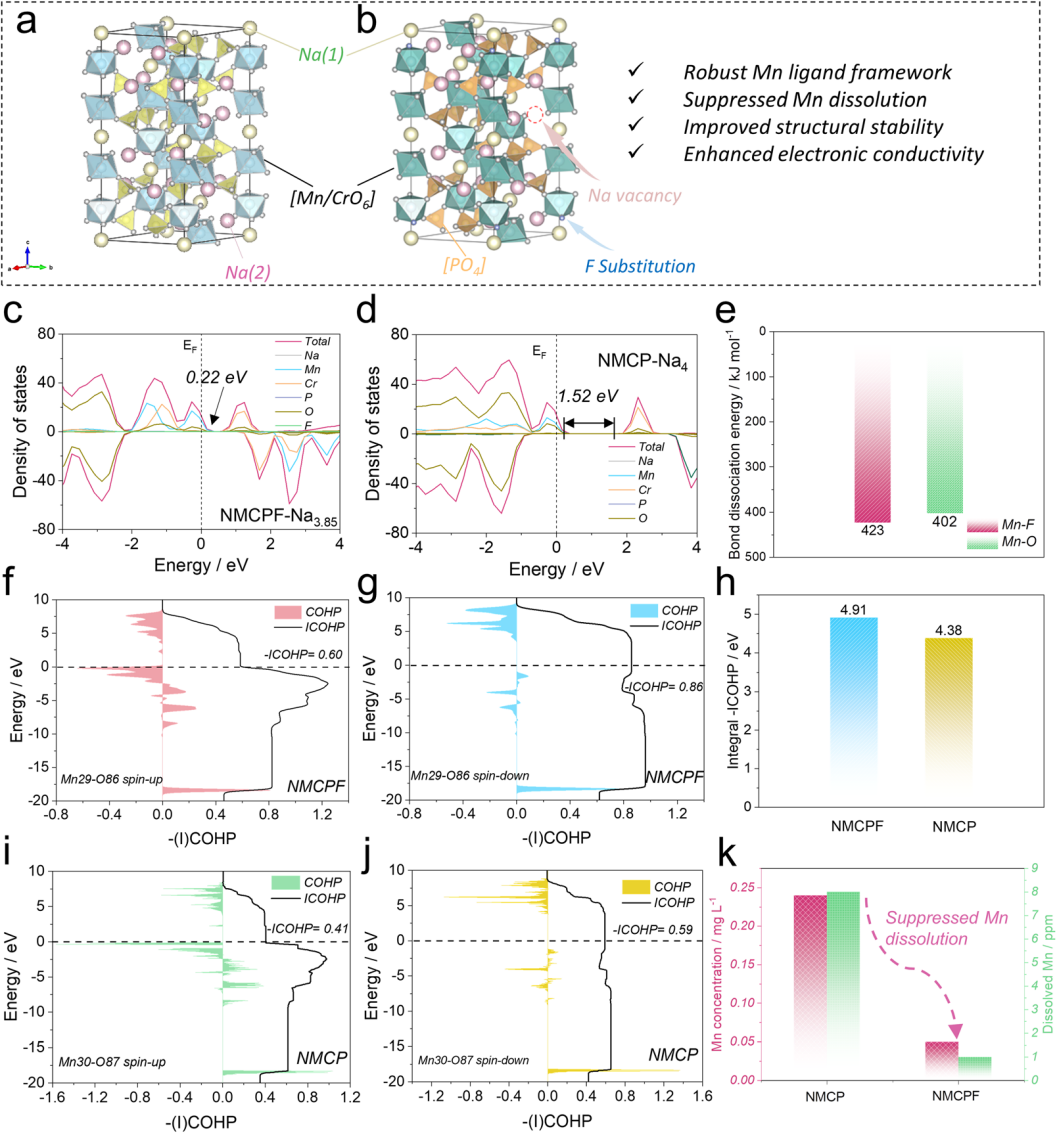
Theoretical studies on the intrinsic physicochemical properties. Structural illustrations of (a) Na_4_MnCr(PO_4_)_3_ and (b) F doped Na_4_MnCr(PO_4_)_3_. Total and partial DOS patterns of (c) NMCPF and (d) NMCP. (e) Bond dissociation energies of Mn–F and Mn–O. (f and g) COHP and ICOHP results of Mn29–O86 bond (spin-up and spin-down) in NMCPF. (h) Integral -ICOHP values of all Mn–O/F bonds in NMCPF and NMCP. (i and j) COHP and ICOHP results of Mn30-O87 bond (spin-up and spin-down) in NMCP. (k) Dissolved Mn concentrations in the electrolytes (claret-red) and separators (green).

As a proof of concept, here in this paper, we report an effective doping strategy *via* introducing a well-controlled amount of light-weight fluorine (F) into Na_4_MnCr(PO_4_)_3_, (denoted as NMCPF; NMCP for the pristine sample). The functions of the light-weight F dopant were deeply studied by both experimental and theoretical approaches, which were subsequently shown to contribute to a “Mn-locking” effect—robust Mn ligand framework, suppressed Mn dissolution, improved structural stability and enhanced electronic conductivity as illustrated in Fig. 1a and b. The comprehensive analyses of *in situ* and *ex situ* measurements confirmed that the F dopant has no impact on structural evolutions (*i.e.*, both solid solution and two-phase reactions are involved) of the original Na_4_MnCr(PO_4_)_3_. It turned out that an excellent rate performance up to 40C and an improved cycling stability (1000 cycles at 20C) were achieved. This work proposes an effective and broadly applicable light-weight F doping strategy for high-performance polyanionic SIBs cathodes without side effects.

## Results and discussion

### Theoretical calculations of the fluorine-doped Mn-rich phosphates

To obtain in-depth insights on the physicochemical properties, we employed density functional theory (DFT) calculations. The bond valence (BV) method has been widely adopted to identify the ionic diffusion pathways within structures as a typical empirical approach. From the BV maps in Fig. S1 (ESI,† the isosurface value is 0.8), both samples possess 3D well-interconnected pathways for facile Na^+^ ions diffusion with the lowest energy regions except for some subtle differences, which is the feature of typical NASICONs.^44,45^ Convex-hull phase diagrams of F-doped Na_4_MnCr(PO_4_)_3_ (NMCPF; Fig. S2a, ESI†) and Na_4_MnCr(PO_4_)_3_ (NMCP; Fig. S2b, ESI†) were constructed hinged on the formation energy at different de-/sodiation states with the structural transformations when cycling. All the metastable phases of Na_4_MnCr(PO_4_)_3_ (or Na_3.85_MnCr(PO_3.95_F_0.05_)_3_) to Na_0_MnCr(PO_4_)_3_ (or Na_0_MnCr(PO_3.95_F_0.05_)_3_) could be electrochemically achieved on account of the negative values of formation energies, which may be the intrinsic origin for multi-electron redox reactions.^35,36^ Of note, Na_2_MnCr(PO_4_)_3_ or Na_1.85_MnCr(PO_3.95_F_0.05_)_3_ emerged as the most stable phase due to the lowest formation energy, which contributed to the relatively good performances between 1.5–4.3 V rather than 1.5–4.5 V *versus* Na^+^/Na.^35,37^

The voltage profiles of NMCPF (Fig. S3a, ESI†) and NMCP (Fig. S3b, ESI†) were also calculated with regard to Mn^2+^/Mn^3+^, Mn^3+^/Mn^4+^ and Cr^3+^/Cr^4+^ redox couples, both of which fitted well with the experimental (galvanostatic intermittent titration technique, GITT) results (with slight deviations).^36^ In general, the calculated voltage platforms of NMCPF were relatively higher than those of NMCP, which may be ascribed to the “inductive effect” by highly electronegative F (3.98 *versus* 3.44 for O).^46^ An ultrahigh voltage plateaus at ∼5 V is presumed to be activated from the calculation if an optimized electrolyte that endures such a high voltage could be developed, which would greatly increase the overall energy density. In addition, the density of states (DOS) diagrams (Fig. 1c and d) were obtained, from which we could find the hybridized Na 3s, Mn 3d, Cr 3d, P 2p, and O 2p orbitals in both samples; one more F 1s orbital existed in NMCPF. It turns out that the forbidden band gap is greatly decreased from 1.52 eV in NMCP to 0.22 eV in NMCPF, which contributes to the enhanced electronic conductivity after F modification. The rest DOS patterns with different Na contents are displayed in Fig. S4 (ESI†). The results of NMCP in Fig. S5a (ESI†) and NMCPF in Fig. S5b (ESI†) further support the modification of charge densities through F doping. These results are favorable for better electrochemical properties of NMCPF from a kinetics perspective.

To further reveal the capacity fading mechanism of the pristine NMCP, we dissembled the cycled batteries in the glove box to collect the electrolyte and the separator and measured the separate manganese concentration through Inductively Coupled Plasma Optical Emission Spectrometer (ICP-OES). Like other Mn-based materials, NMCP also suffers from severe Mn dissolution with the Mn concentrations of 0.24 mg L^−1^ in the electrolyte and 8 ppm in the separator after cycling (Fig. 1k), which could account for the inferior electrochemical performances of NMCP.^39,47^ In sharp contrast, the manganese dissolution issue in NMCPF was effectively suppressed with a low Mn concentration of 0.05 mg L^−1^ in the electrolyte and 1 ppm in the separator after cycling. In addition, we employed a theoretical approach to explain this phenomenon. Crystal Orbital Hamilton Population (COHP) has been acknowledged as a reliable tool to visualize the chemical bonding in the battery field recently.^23,48,49^ The Kohn–Sham states were initially projected into atomic orbitals and subsequently the mutual orbital overlap population was inspected. The COHP and ICOHP (integrated COHP) results are shown in Fig. 1f, g, i, j, S6, S7 (ESI), 1h, Tables S1and S2 (ESI†) based on the adjacent six O/F atoms around a specific Mn atom. As reported, the negative value of COHP of a Mn–O/F pair represents constructive (namely, bonding) interference of atomic orbitals; the modulus of COHP corresponds to the degree of covalency between Mn and O/F and the zero point of COHP suggests non-interacting bonds or a pure ionic bond. In each pattern of Fig. 1f, g, i and j, S6 and S7 (ESI†), the intersection between ICOHP curve and Fermi level is the actual -ICOHP value. From Fig. 1h, NMCPF possesses a higher integral -ICOHP value (all six Mn–O/F pairs including spin-up and spin-down direction) of 4.91 eV than NMCP (4.38 eV). This result indicates that F doping strengthens the overall chemical bonding of Mn–O/F bonds in the local scope, which may be associated with the high electronegativity of F and the higher bond dissociation energy of Mn–F (423 kJ mol^−1^) than that of Mn–O (402 kJ mol^−1^) in Fig. 1e.^50,51^ The strengthened chemical bonding between adjacent O/F and Mn triggers a “Mn-locking” effect in NMCPF that suppresses manganese dissolution and thus improved electrochemical properties could be obtained as illustrated in Fig. 1a and b.

### Structural analysis of materials

**Fig. 2:**
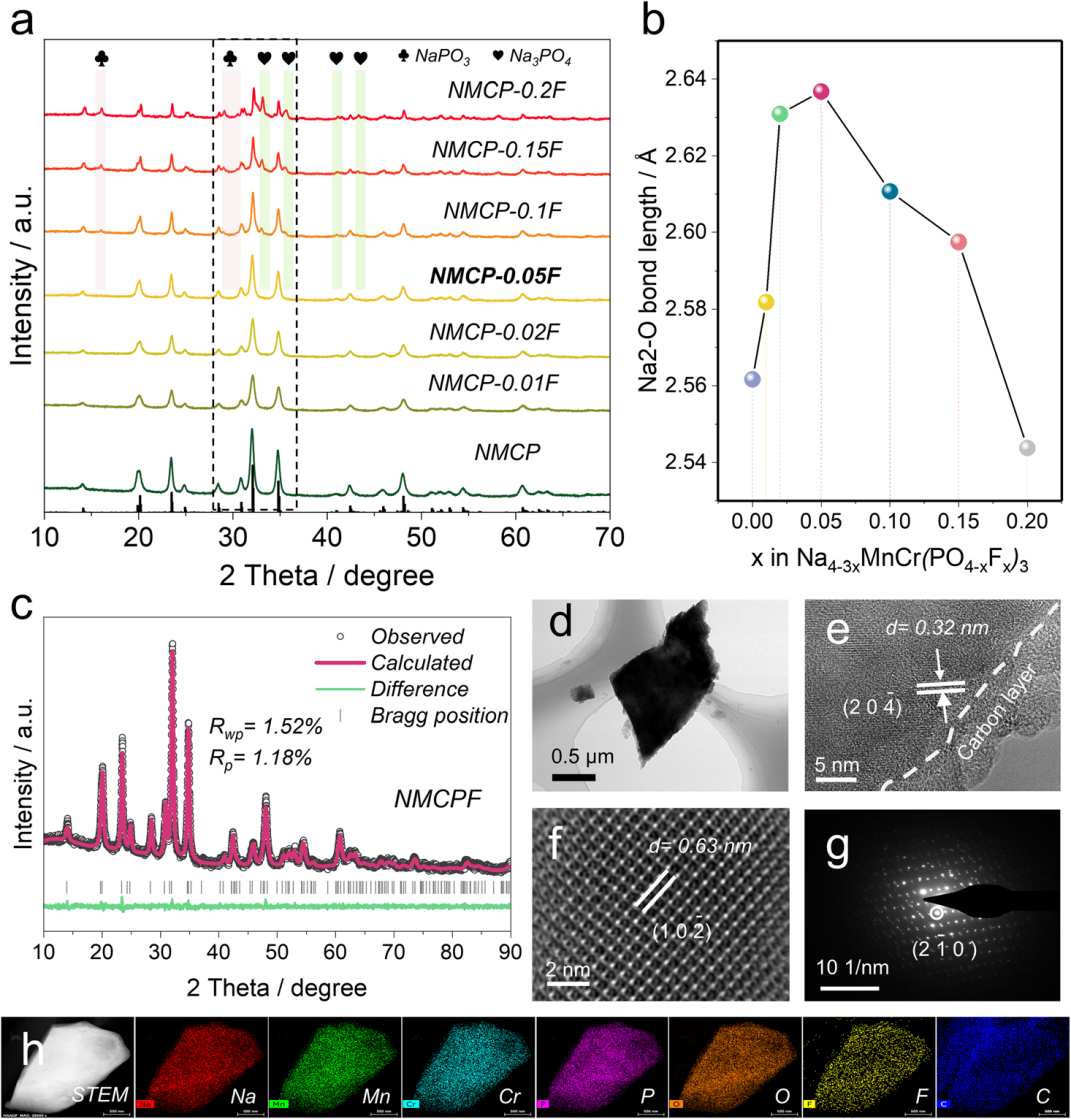
Materials characterizations. (a) Comparison of X-ray diffraction (XRD) results of different as-prepared samples. (b) Bond lengths of Na(2)–O within different as-prepared samples. (c) XRD Rietveld refinement of NMCPF. (d) TEM, (e) HRTEM and (f) HAADF-STEM images of NMCPF. (g) SAED pattern of NMCPF. (h) EDS mapping results of NMCPF (scale bar: 500 nm).

The designed NASICON materials were synthesized by a simple sol–gel method. The X-ray diffraction (XRD) results (NMCP, NMCP-0.01F, NMCP-0.02F, and NMCP-0.05F) in Fig. 2a present analogous patterns, which determined a pure Na_4_MnCr(PO_4_)_3_ phase within these samples and proved the partial introduction of F had no impact on the structure within this range of F contents (*x* = 0–0.05). Notedly, NaPO_3_ (JCPDS#00-003-0688) and Na_3_PO_4_ (JCPDS#00-030-1232) impurity phases appear with *x* value higher than 0.05.^52^ In Fig. S8 (ESI†), it can be clearly found that the rate properties improved initially with an increased F ratio (*x* = 0, 0.01, 0.02, 0.05) and reached a peak value at *x* = 0.05 (NMCP-0.05F), accompanied by a descending trend when further increasing *x*. To disclose the underling mechanism, the bond lengths of Na(2)–O were calculated (Fig. 2b). At the initial stage, there was an increasing trend with a maximum Na(2)–O distance of 2.637 Å (*x* = 0.05) and then the distance decreased with higher fluorine contents. According to previous works, longer the bond length of Na(2)–O (weaker bond strength) is associated with faster Na^+^ diffusion, which explains the performance discrepancy in Fig. S8 (ESI†).^32,53^ Therefore, the material with *x* = 0.05 (NMCP-0.05F) was selected as the optimal one (NMCPF; the baseline sample (*x* = 0) is denoted as NMCP).

XRD Rietveld refinements determined that both NMCPF (Fig. 2c) and NMCP (Fig. S9, *x* = 0, ESI†) were indexed to the *R*3̄*c* space group with low *R*_wp_ values of 1.52% and 1.48%, respectively. The detailed structural information is listed in Tables S3 and S4 (ESI†). Fig. 1a and b present an illustration of their crystalline structures. Both samples crystalized in a similar trigonal NASICON structure: Cr and Mn atoms share the same 12*c* site with 50% fraction for each position; [PO_4_] or [PO_3.95_F_0.05_] tetrahedra are corner-shared with [CrO_6_] or [MnO_6_] octahedra to construct the lantern-like [MnCr(PO_4_)_3_] or [MnCr(PO_3.95_F_0.05_)_3_] constituents, and thus two Na^+^ host sites are formed. Na(1) (6*b* site) is sixfold coordinated while the other Na(2) (18*e* site) is eightfold coordinated which tends to be more easily extracted/inserted from/to the NASICON structure due to the weaker bonding force of Na(2)–O.^30^ As anticipated, F atoms partially occupy in O1 and O2 sites with the fractions of 1.2% for each position from Table S4.† According to the valence-induced mechanism, the less negative charge stemming from the substitution of O^2−^ by F^−^ should be compensated by cation deficiency (*i.e.*, Na^+^ deficiency) to form Na_3.85_MnCr(PO_3.95_F_0.05_)_3_.

ICP-OES results further determined the chemical formula. As shown in Fig. S10 (ESI†), the atomic ratios were measured to be Na(3.84), Mn(1), Cr(1.01) and P(2.95) for NMCPF and Na(3.96), Mn(1), Cr(1.01) and P(2.97) for NMCP, which echoes well with the above discussions. These results indicate that some Na vacancies exist in NMCPF as illustrated in Fig. 1a and b. Transmission electron microscopy (TEM) energy dispersive spectrometry (EDS) mapping images of NMCPF (Fig. 2h) and NMCP (Fig. S11d, ESI†) present a uniform distribution of all elements, which indicate the successfully introduction of F into NMCPF. Solid-state nuclear magnetic resonance (NMR) spectroscopy of ^19^F was performed to further study the F substitution. The broad resonance of −80–−180 ppm in Fig. S12 (ESI†) determined that F atoms partially substituted O atoms in NMCPF.^42,43,54^ The above results validate that O atoms were partially occupied by F atoms within NMCPF.

Furthermore, TEM was employed to probe the microstructures. As displayed in Fig. 2d–e, S11a and b (ESI†), both particles are homogenously coated with an amorphous carbon layer of 2–5 nm. Similar irregular morphologies as they displayed, the size of NMCPF (0.5–1 μm) is smaller than that of NMCP (1–2 μm). Besides, high-resolution (HR) TEM images (Fig. 2e and S11b, ESI†) show a lattice fringe of 0.32 nm, which corresponds to the (2 0 4̄) lattice plane. HAADF-STEM image in Fig. 2f demonstrates the high crystallinity within NMCPF with apparent atomic distribution. The selected area electron diffraction (SAED) in Fig. 2g and S11c (ESI†) further determined the well-crystallized NMCPF and NMCP. Besides, it can be clearly observed from SEM images in Fig. S13c and d (ESI†) that NMCP appears an irregular morphology and the micron particles. When introducing F into NMCP, NMCPF presents apparent hierarchical structure of various nanoparticles (Fig. S13a and b, ESI†), which are relatively smaller than those of NMCP. These results correspond well to TEM images of Fig. 2d, h, S11a and d (ESI†). Furthermore, both samples display a relatively smooth surface except that they are covered by some small particles (Fig. S13b and d, ESI†).

As shown in Fig. S14 (ESI†), the amount of carbon was measured to be 10.85% for NMCPF and 11.61% for NMCP by the thermogravimetric analysis (TGA). In Fig. S15 (ESI†), identical Fourier transform infrared (FT-IR) spectra of NMCPF and NMCP clearly shown the stretching or bending vibration signals of [CrO_6_] or [MnO_6_] octahedra and [PO_4_] tetrahedra.^37,55^ X-ray photoelectron spectroscopy (XPS) results (Fig. S16, ESI†) display the existence of Na, Cr, Mn, P, O and C for both NMCPF and NMCP. But it is of note that F only exists in NMCPF and the F 1s has a characteristic peak at 683.93 eV. In contrast, there are no distinguishable signals of F in NMCP.

### Structural evolution

**Fig. 3:**
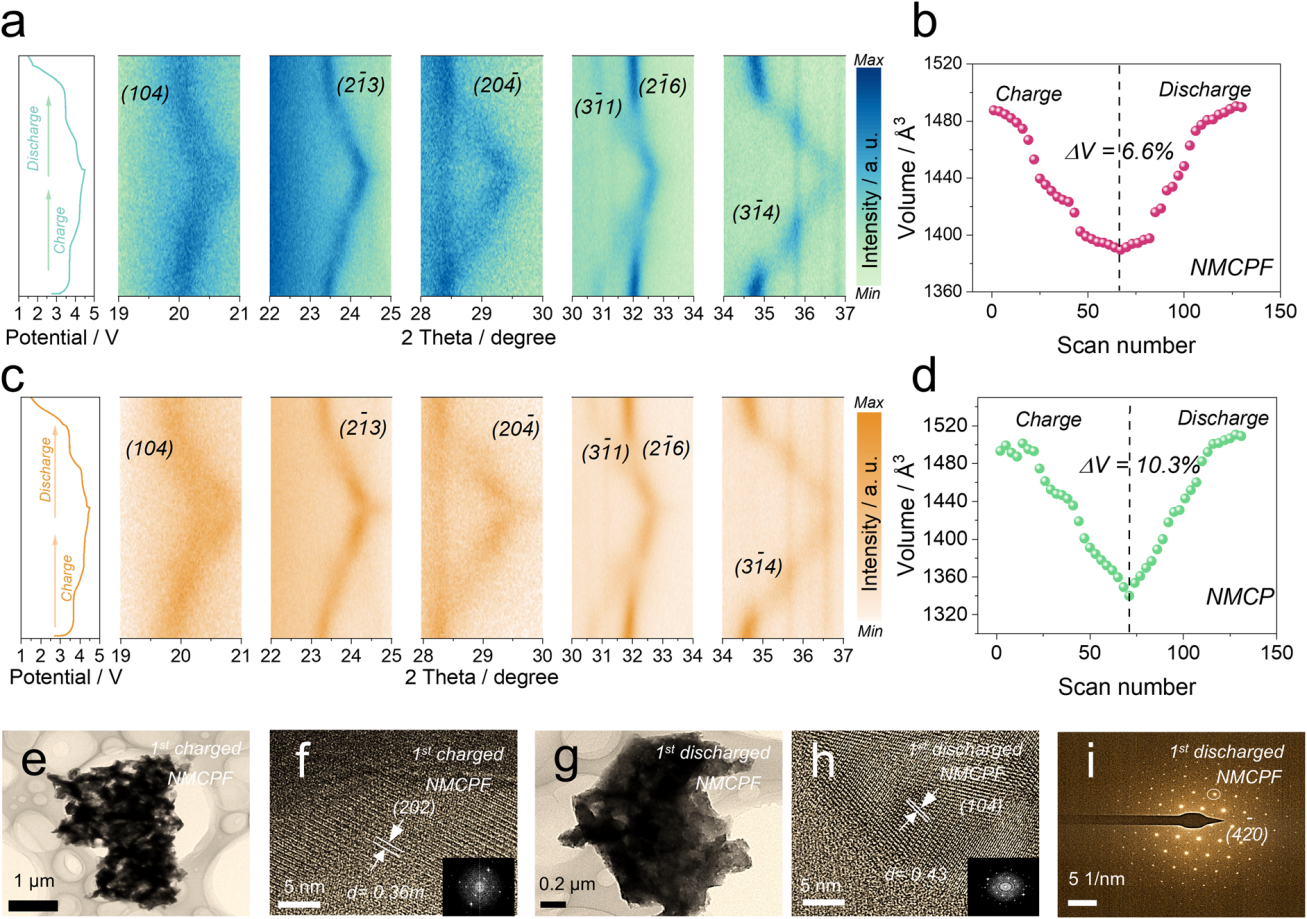
Structural evolution. (a, b) *In situ* XRD contour patterns and corresponding changes of the lattice volume of NMCPF. (c, d) *In situ* XRD contour patterns and corresponding changes of the lattice volume of NMCP. (e, f) TEM and HRTEM images (inset: FFT pattern) of the 1^st^ charged NMCPF. (g–i) TEM, HRTEM images (inset: FFT pattern) and the SAED pattern of the 1^st^ discharged NMCPF.

Aiming to unravel the structural evolutions and variation of sodium-ion storage mechanism in NMCP after F doping, *in situ* XRD tests were performed within 1.5–4.5 V (*vs.* Na^+^/Na) for one cycle (left sides of Fig. 3a and c) by a commercially designed electrolytic cell (Beijing SciStar technology Co., Ltd) with a beryllium window under a relatively low current density (0.1C). Because a high current treatment may lead to a large polarization that will influence the accuracy of structural information (we need to collect more data points as best as we can to realistically reflect the real conditions upon battery cycling within a XRD scan loop).^35,56–58^ Fig. S17 (ESI†) depicts the full-scope patterns. Fig. 3a and c clearly showed the reflections of (1 0 4), (2 1̄ 3), (2 0 4̄), (3 1̄ 1), (2 1̄ 6) and (3 1̄ 4) and all of them were highly reversible, which can be ascribed to their robust NASICON structures and contribute to the good reversibility of electrochemical properties that will be discussed later. At first upon charging to ∼3.65 V, all reflections underwent a right-shift, which corresponded to a solid–solution reaction. It should be noted that (2 0 4̄) reflection was split into two peaks from the end of ∼3.65 V; both (3 4̄ 1) and (3 1̄ 4) vanished during the first voltage platform stage corresponding to the oxidation process from Mn^2+^ to Mn^3+^,^36,37^ which confirmed that a second phase appeared with coexistence of the initial phase. At the end of the first voltage plateau, all vanished reflections appeared again. When charging to high voltage (3.72 V–4.5 V), all the peaks gradually shifted to higher 2*θ* values, corresponding to solid-solution reactions when Mn^3+^ is oxidized to Mn^4+^ followed by the oxidation of Cr^3+^ to Cr^4+^.^36^ During discharging, all reflections underwent completely obsequent processes and returned to the initial positions. Therefore, the above results revealed a combination of solid-solution reactions and two-phase reactions during extraction/intercalation of Na^+^ for both samples; while solid-solution reactions were in the majority, which ensure the good reversibility and fast kinetics for Na^+^ storage. The residual Na^+^ in the structure at deep charged states are deemed to stabilize the overall framework as the binding pillars. The above results confirmed that F doping has no impact on Na^+^ storage mechanisms for NMCP.

In addition, we collected the lattice volumes at all stages. As displayed in Fig. 3b and d, two samples underwent analogous transitions: at the beginning upon charging, *v*-axis decreased, corresponding to the structural shrinkage; upon discharging, *v*-axis experienced the reverse processes and finally returned to their initial states, supportive of good reversibility for both samples. Accordingly, the volume change of NMCPF (6.6%) was much lower than that of NMCP (10.3%), which strongly confirmed that F dopant is conducive to improve structural stability. This may be due to the high electronegativity of F and the increased F–metal interaction as discussed in Fig. 1. The extent of peak deviation for NMCPF (Fig. 3a) was also smaller than that of NMCP (Fig. 3c), which coincides well with the results in Fig. 3b and d. The *ex situ* TEM and SAED results of fully charged and discharged NMCPF cathodes are shown in Fig. 3e–i. Some obvious lattice fringes and diffraction spots could be clearly identified, demonstrating that the well-crystallized structure was maintained when even subjected to a deep charge state. Such an enhanced structural stability guarantees the boosted cyclabilities of NCMPF after F doping.

### Electrochemical performance

**Fig. 4:**
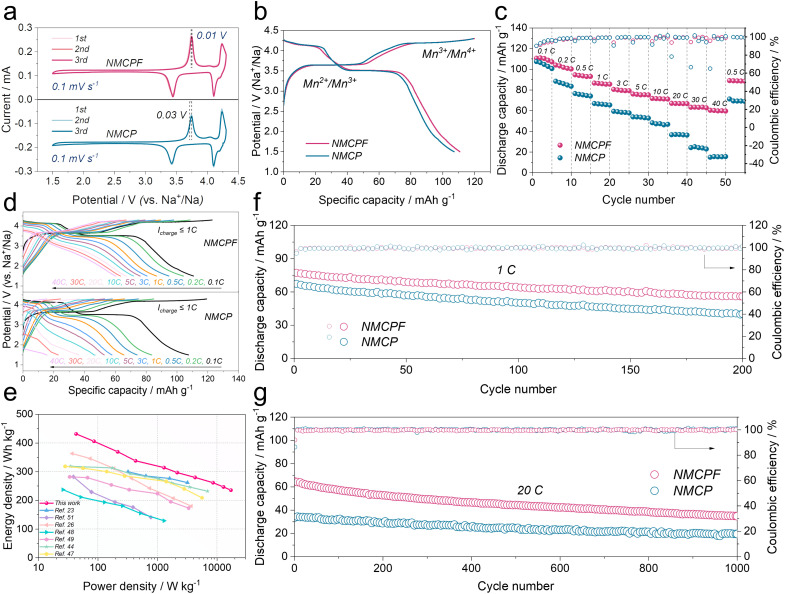
Electrochemical capabilities within 1.5–4.3 V. (a) CV curves at 0.1 mV s^−1^. (b) Initial charge/discharge curves at 0.1C (1C = 110 mA h g^−1^). (c) Rate properties. (d) Charge/discharge profiles at various rates. (e) Ragone plot. Cycling capabilities at (f) 1C and (g) 20C.

The CR2032 type coin cells with metallic Na anodes were fabricated to evaluate the effectiveness of our strategy. In Fig. 4a, two redox couples of 3.75/3.44 V (Mn^2+^/Mn^3+^) and 4.24/4.10 V (Mn^3+^/Mn^4+^) could easily be found in the cyclic voltammetry (CV) curves.^37^ The highly overlapped profiles indicated an excellent reversibility of de-/sodiation processes for both NMCPF and NMCP.^59^ And the narrowed potential gap of NMCPF (0.01 V; 0.03 V for NMCP) showed an improved kinetics with F dopant. In Fig. 4b, two distinguishable plateaus located at 3.65 V and 4.2 V could be observed as well, thus delivering 110.7 mA h g^−1^ (NMCPF) and 107.1 mA h g^−1^ (NMCP) at 0.1C accompanied by successive phase transformations of Na_3.85_MnCr(PO_3.95_F_0.05_)_3_ ↔ Na_2.85_MnCr(PO_3.95_F_0.05_)_3_ ↔ Na_1.85_MnCr(PO_3.95_F_0.05_)_3_ and Na_4_MnCr(PO_4_)_3_ ↔ Na_3_MnCr(PO_4_)_3_ ↔ Na_2_MnCr(PO_4_)_3_, respectively.^36,37^

Rate properties are also pivotal for practical applications. As displayed in Fig. 4c, NMCPF showed much enhanced rate performances compared to NMCP while they delivered analogous initial capacities at 0.1C: 110.6 mA h g^−1^ for NMCPF and 107.5 mA h g^−1^ for NMCP. Increasing current densities from 0.1C to 0.2C, 0.5C, 1C, 3C, 5C, 10C, 20C and 30C, the enlarged capacity gaps were observed. Notably, even at 40C, NMCPF still delivered 60.4 mA h g^−1^, which outweighs all of previous reports on Na_4_MnCr(PO_4_)_3_.^35–37,60^ However, NMCP only revealed a “NEAR ZERO” capacity of 15 mA h g^−1^ at 40C. When back to 0.5C, a capacity of 89.1 mA h g^−1^ for NMCPF was still recovered, demonstrative of the remarkable reversibility. Moreover, Fig. 4d proved a restrained electrochemical polarization of NMCPF.^59^Fig. 4e also compared the performances of NMCPF with other reported cathode materials hinged in Na half-cells. It turns out that NMCPF with higher energy densities surpassed the majority of the proposed materials.

Cycling stabilities under various current densities are important as well. In Fig. S18 (ESI†), NMCPF showed an initial capacity of 110.8 mA h g^−1^ at 0.1C; it could stably operate for 65 cycles. Nevertheless, NMCP possessed a much poorer cyclability and only a low capacity of 81.5 mA h g^−1^ could be maintained after 65 cycles under the identical condition. Fig. 4f (at 1C), Fig. S19 (at 0.5C; ESI†) and Fig. S20 (at 5C; ESI†) showed apparently improved capacity retentions and cycling stabilities for NMCPF in comparison to NMCP. As displayed in Fig. S21 (ESI†), we also subjected the batteries to a higher rate of 10C to investigate their long-term cyclabilities. Markedly, NMCPF could steadily cycle 500 times at 10C (Fig. S21, ESI†) and work for 1000 cycles at 20C (Fig. 4g) with boosted coulombic efficiencies. In contrast, capacity retentions of merely 56.7% at 10C and 51.3% at 20C were achieved for NMCP. The cycling performances of NMCPF also surpass those of NMCP and many other reported cathode materials (Table S5, ESI†).

In addition, the batteries were tested within a wider voltage window of 1.5–4.5 V to further determine the merits of our work. In Fig. S22a and b (ESI†), both the CV profiles and charge–discharge curves exhibit three pairs of redox couples and three apparent voltage platforms. Apart from Mn^2+^/Mn^3+^ and Mn^3+^/Mn^4+^, a high-potential Cr^3+^/Cr^4+^ (4.4 V) was also activated in these two samples thereby 143 mA h g^−1^ for NMCPF and 133.6 mA h g^−1^ for NMCP were obtained, which coincides with previous works.^35,36,60^ Furthermore, rate performances (Fig. S22c, ESI†) and the cycling stability (Fig. S22d, ESI†) of NMCPF were better than those of NMCP. Further improvements rely on the development of the novel high-voltage electrolyte to endure such a harsh cut-off voltage (Fig. S23, ESI†).

Therefore, the above results confirmed the effectiveness of the F doping strategy on NMCPF. Two typical approaches were carried out to further evaluate the Na^+^ diffusion coefficients (*D*_Na_^+^). The first one is hinged on galvanostatic intermittent titration technique (GITT) method (Fig. S24a, ESI†) after five activation cycles. As seen in Fig. S24b (ESI†), NMCPF displayed higher *D*_Na_^+^ (10^−13^ to 10^−8^ cm^2^ s^−1^) than NMCP (10^−14^ to 10^−8^ cm^2^ s^−1^), which indicates the enhanced Na^+^ kinetics after F doping. Also, Fig. S24c (ESI†) showed suppressed overpotentials for NMCPF. Likewise, the batteries were subjected to 0.1–1.0 mV s^−1^ under CV tests (Fig. S26, ESI†). The linear fitting profiles of Fig. S26b (ESI†) and Fig. S26d (ESI†) reveal diffusion-controlled processes during Na^+^ extraction/intercalation for both NMCPF and NMCP.^61^ Accordingly, NMCPF showed higher *D*_Na^+^_ values than NMCP in Fig. S24d (ESI†) based on the Randles–Sevcik equation. This enhancement could be on account of the construction of Na vacancies due to F substitution, which truly facilitated Na^+^ diffusion. Electrochemical impedance spectra (EIS) results in Fig. S27† further support the reduced charge transfer impedance, which corresponds to the decreased forbidden band gap in Fig. 1c, d and S4 (ESI†).

### Full cell evaluation

**Fig. 5:**
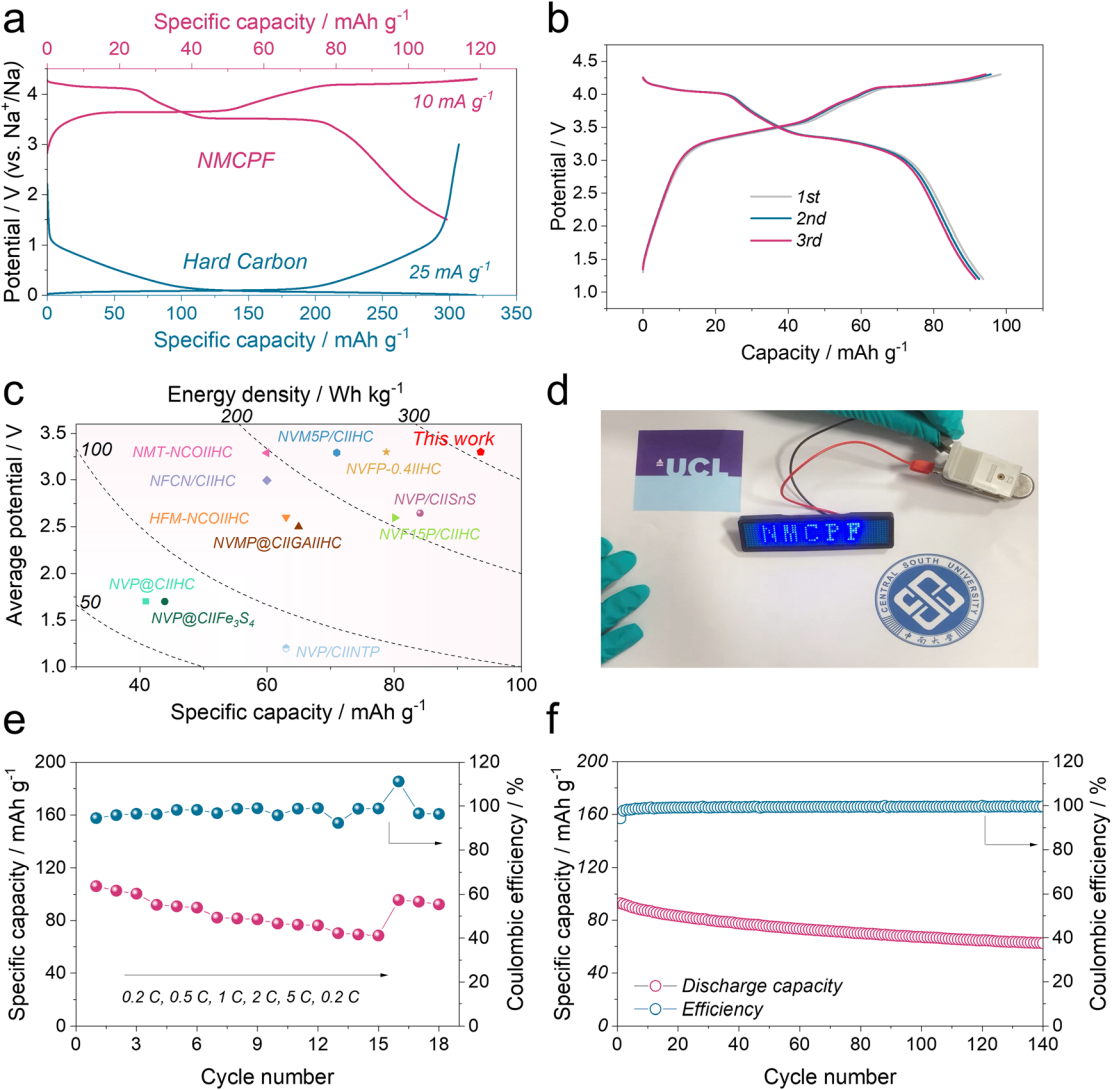
Electrochemical performances of the HC‖NMCPF full cell. (a) Voltage curves of the HC anode and the NMCPF cathode. (b) Charge–discharge profiles of the HC‖NMCPF full cell for the initial three cycles at 0.5C (1C = 110 mA g^−1^). (c) Comparison of our work with previous reported works. (d) Picture of a LED badge powered by the HC‖NMCPF full cell. (e) Rate capability of the full cell. (f) Cycling stability of the full cell at 0.5C.

With practical application concerns, the NMCPF cathode was coupled with commercial hard carbon (HC) anode to construct full cells (HC‖NMCPF). To fabricate a high-performance full cell, prior to the full cell assembly, the HC electrodes were initially pre-cycled in a half-cell configuration with Na metal anodes at 25 mA g^−1^ (Fig. S28, ESI†) to avoid the Na^+^ loss of the cathode due to the SEI layer formation according to previous works.^36,37,62^ The pre-sodiated HC was then taken out at the discharge state after 5 cycles. The mass ratio of the NMCPF cathode to the HC anode was carefully controlled to be 2.2 : 1 to balance the capacity. It delivered a typically reversible capacity of ∼316 mA h g^−1^ with a low voltage platform of ∼0.1 V (*versus* Na^+^/Na). The mass ratio of the NMCPF cathode to the HC anode was carefully controlled to be 2.2 : 1 to balance the capacity as illustrated in Fig. 5a. Within the voltage window of 1.2–4.3 V, the galvanostatic charge–discharge profiles of HC‖NMCPF at 0.5C were presented in Fig. 5b, in which a high initial capacity of 93.6 mA h g^−1^ was obtained with two discernible voltage plateaus, in line with Fig. 4. Besides, the HC‖NMCPF full cell was capable of powering a LED badge (Fig. 5d). Based on the total mass of cathode and anode, a capacity of 93.6 mA h g^−1^ along with an average voltage of 3.3 V was achieved, which corresponds to a high energy density of ∼309 W h kg^−1^. This result outweighs those of most full cells (Fig. 5c). Furthermore, good rate capabilities from 0.2C to 5C and outstanding cycling stability at 0.5C were realized as well (Fig. 5e and f). The above full cell properties further confirmed the advantage of this work.

## Conclusions

In summary, we have proposed an effective modification strategy of well-controlled doping light-weight F into the Na_4_MnCr(PO_4_)_3_ lattice. An enhanced cycling stability (1000 cycles at 20C) and an excellent rate performance up to 40C were thus obtained. The good electrochemical performances could be attributed to the reduced energy gap (the boosted electronic conductivity) and strengthened local chemical bonding around Mn as confirmed by DFT calculations. The ICP-OES results revealed suppressed Mn dissolution after the modification of F. The structural transformations (mainly for the solid solution reaction and partially for the two-phase reaction) remained unchanged as determined by *in situ* XRD and TEM. This research proves the prospect of light-weight F doping for high-voltage polyanionic cathodes for SIBs technology.

## Data availability

The data that support the findings of this study are available from the corresponding author upon reasonable request.

## Author contributions

W. Z. conceived the idea and lead this work. W. Z. and Y. W. synthesized the materials and carried out all the electrochemical measurements. Z. X. performed the DFT calculations. Y. W., Y. D., L. H., Z. L. and S. L. conducted the XRD, XPS and NMR studies. R. C., X. G., W. Z., F. G., J. Z., H. D. and J. L. contributed to the *in situ* XRD, ICP, TEM and Raman tests. Z. Z., G. H., Y. L. and I. P. P. supervised the project and co-wrote the manuscript. All authors discussed the results and contributed to writing the manuscript.

## Conflicts of interest

The authors declare no competing interests.

## Supplementary Material

SC-014-D3SC03095D-s001
