# Supplementary material for: “Mn-locking” effect by anionic coordination manipulation stabilizing Mn-rich phosphate cathodes
Source: Chem Sci. 2023 Jul 27;14(32):8662–71. doi: 10.1039/d3sc03095d (PMC10430554; doi:10.1039/d3sc03095d)
Supplement: SC-014-D3SC03095D-s001 [file SC-014-D3SC03095D-s001.pdf]

Electronic Supplementary Material (ESI) for Chemical Science.
This journal is © The Royal Society of Chemistry 2023

Electronic Supplementary Material (ESI) for Chemical Science
This journal is © The Royal Society of Chemistry 2023

"Mn-locking" effect by anionic coordination manipulation stabilizing Mn-Rich Phosphate Cathodes

Wei Zhang,‡[abc] Yulun Wu,‡[a] Yuhang Dai,[c] Zhenming Xu,*[d] Liang He,[a] Zheng Li,[a] Shihao Li,[a] Ruwei Chen,[b] Xuan Gao,[b] Wei Zong,[b] Fei Guo,[c] Jiexin Zhu,[c] Haobo Dong,[c] Jianwei Li,[b] Chumei Ye,[e] Simin Li,[a] Feixiang Wu,[a] Zhian Zhang,*[a] Guanjie He,*[bc] Yanqing Lai,*[a] and Ivan P. Parkin,*[b]

[a] School of Metallurgy and Environment, Engineering Research Center of the Ministry of Education for Advanced Battery Materials, Hunan Provincial Key Laboratory of Nonferrous Value-Added Metallurgy, Central South University, Changsha 410083, P. R. China

[b] Christopher Ingold Laboratory, Department of Chemistry, University College London, London WC1H 0AJ, UK

[c] Electrochemical Innovation Lab (EIL), Department of Chemical Engineering, University College London, London WC1E 7JE, UK

[d] Jiangsu Key Laboratory of Electrochemical Energy Storage Technologies, College of Materials Science and Technology, Nanjing University of Aeronautics and Astronautics, Nanjing 210016, P. R. China

[e] Department of Materials Science and Metallurgy, University of Cambridge, Cambridge CB3 0FS, UK

‡ These authors contributed equally.

*E-mail: xuzhenming@nuaa.edu.cn; zhangzhian@csu.edu.cn; g.he@ucl.ac.uk; laiyanqing@csu.edu.cn; i.p.parkin@ucl.ac.uk

Methods

1. Materials synthesis

A typical sol-gel approach was performed to synthesize 5 mmol $Na_{4-3x}MnCr(PO_{4-x}F_x)_3$, where x = 0, 0.01, 0.02, 0.05, 0.1, 0.15 and 0.2 (denoted as NMCP, NMCP-0.01F, NMCP-0.02F, NMCP-0.05F, NMCP-0.1F, NMCP-0.15F and NMCP-0.2F). All chemicals were directly used without further purification. Anhydrous citric acid (≥99.5%, Sigma-Aldrich), $(CH_3COO)_2Mn{\cdot}4H_2O$ (≥99.0%, Sigma-Aldrich), $Cr(NO_3)_3{\cdot}9H_2O$ (≥99.0%, Sigma-Aldrich), CH_3COONa (≥99.0%, Sigma-Aldrich), NaF (≥99.0%, Sigma-Aldrich) and $NH_4H_2PO_4$ (≥99.5%, Sigma-Aldrich) with the designed ratios were successively dissolved into an aqueous solution (60 mL ultrapure water). The molar ratio of citric acid to transition metals was controlled as 3:2. Afterwards, the solution was heated at 80 °C accompanied by constant magnetic stirring for 5 h and then was kept in a 120 °C oven overnight. After that, the dry solid precursor was grinded into fine powders and further subjected to 650 °C for 9 h under argon atmosphere.

2. Physicochemical characterizations

The XRD patterns were collected on a PANalytical Empyrean device and the lattice parameters were refined with a GSAS software by Rietveld method.[1, 2] VESTA software was adopted to depict the structure schematic illustration.[3] A LabRAM HR Evolution instrument (laser wavelength: 532 nm) was carried out to collect Raman spectra. XPS spectra were obtained on a ThermoFisher ESCALAB 250Xi facility (Al K_α radiation) and were calibrated based on C 1s (284.8 eV). The binding energies of peaks were referred to the National Institute of Standards and Technology (NIST, USA) database. TGA was performed on a STA 2500 Regulus device (heating rate: 10 °C min^{-1}) under ambient atmosphere. The TEM images of the samples were taken on Titan G2 60-300. ICP tests were conducted on Spectro Blue SOP. The *in-situ* XRD patterns were obtained by a well-designed cell with an X-ray penetrable Be window that also serves as the current collector. The cycled electrodes were disassembled in a glove box and were thoroughly rinsed by dimethyl carbonate solvent before further use.

3. Battery assembling and measurements

The cathode consists of NMCPF/NMCP, acetylene black and polyvinylidene fluoride with a typical ratio of 7:2:1 (N-methyl-2-pyrrolidone as the solvent). The slurry was then uniformly pasted onto the surface of Al foil by a doctor blade. Afterwards, the wet electrode was further dried at 80 °C for 12 h within a vacuum oven. The batteries (CR2032 coin cell) were assembled in a pure-argon glove box with a low O_2 and H_2O concentration (< 0.1 ppm). The glass fiber (Whatman GF/D) served as the separator. Na metallic disks were counter electrodes. 1.0 M $NaClO_4$ in propylene carbonate (PC) and ethylene carbonate (EC) (1:1 vol.%) solvents with 5 vol.% fluoroethylene carbonate (FEC) additives served as the electrolyte. Neware battery test system (CT-4008T-5V10mA-164, Shenzhen, China) was adopted to measure the electrochemical performances and GITT data. Five formation cycles were conducted prior to GITT tests. CV and EIS tests were performed on a Biologic VMP-3 electrochemical workstation. The commercial hard carbon was acquired from KURARAY Co., Ltd., Japan. The mass loading of the cathode for half cells was ≈2.0 mg cm^{-2}. As for full cells, to balance the capacity controlling the active mass ratio of cathode to anode as 2.2:1, the mass loading of the cathode was ≈3.0 mg cm^{-2} and that of the anode was ≈1.4 mg cm^{-2}. The N/P ratio of 1.1 was regulated with reference to the corresponding reversible capacities and the current rate was applied with reference to the cathode.

4. Computational methods

All calculations were conducted via the projector augmented wave method in the framework of the density functional theory (DFT),[4] as implemented in the QUANTUM ESPRESSO.[5] The generalized gradient approximation (GGA) and Perdew–Burke–Ernzerhof (PBE) exchange functional[4] were used. The plane-wave energy cutoff was set to 38 Ry, and the Monkhorst–Pack method[6] was employed for the Brillouin zone sampling. The convergence criteria of energy and force calculations were set to 10^{-5} Ry/atom and 0.01 Ry/Å, respectively. To consider the strong correlation effects of transition metal in $Na_{4-x}MnCr(PO_4)_3$ and $Na_{3.85-y}MnCr(PO_{3.95}F_{0.05})_3$, both structural optimizations and electronic structure calculations were carried out by using the spin-

dependent GGA plus Hubbard correction U (GGA + U) method,[7] and the effective U_{eff} parameters are 3.9 and 3.7 eV for Mn-3d and Cr-3d states,[8] respectively. $Na_4MnCr(PO_4)_3$ is built from $Na_4Mn(PO_4)_3$ with equal molar of Mn-Cr mixing, that is Mn-Cr cation arrangements in the Mn sites were created within the conventional cell of $Na_4Mn(PO_4)_3$ using the enumeration method[9] implemented in the Pymatgen code.[10] The Mn-Cr cation arrangement of the most stable $Na_4MnCr(PO_4)_3$ structure was determined by ranking the Mn-Cr cation arrangements by their DFT energies. Furthermore, determination of the stable structures of the sodium extracted compounds with Na-vacancy mixing, including $Na_3MnCr(PO_4)_3$, $Na_2MnCr(PO_4)_3$ and $NaMnCr(PO_4)_3$, was employed through this same method. Similarly, the most stable F-doped $Na_{3.85}MnCr(PO_{3.95}F_{0.05})_3$ structure was constructed by enumerating O anion sites in $Na_4MnCr(PO_4)_3$ structure and ranking their DFT energies. The bond-valence sum (BVS) mappings were performed by the PyAbstantia code. The crystal orbital Hamilton population (COHP) between neighboring oxygen ions was computed by the Lobster program,[11] in which the negative and positive COHP values indicate bonding and anti-bonding, respectively. The pbeVaspFit2015 basis sets were used in the reconstruction of the PAW wave functions of each element. During the charging and discharging of an alkali-ion battery, an alkali A is intercalated or deintercalated from a host crystal structure A_nH. For a battery that operates by shuttling *x* A^+ ions between the cathode and a pure alkali metal anode, the overall cell reaction can be written as follows:

$$A_{n-x}H\ (s) + xA\ (s) = A_nH\ (s)$$

where the typical phase of each compound is indicated for clarity. The forward reaction is the cell discharging reaction, while the reverse is the cell charging reaction. The average intercalation potential V vs. A/A^+ can then be calculated using the following expression:[12]

$$V = -\frac{E(A_nH) - E(A_{n-x}H) - xE(A)}{xe}$$

where E is the total energy as calculated using DFT, and e is the absolute value of the electron charge.

Supplementary Figures and Tables

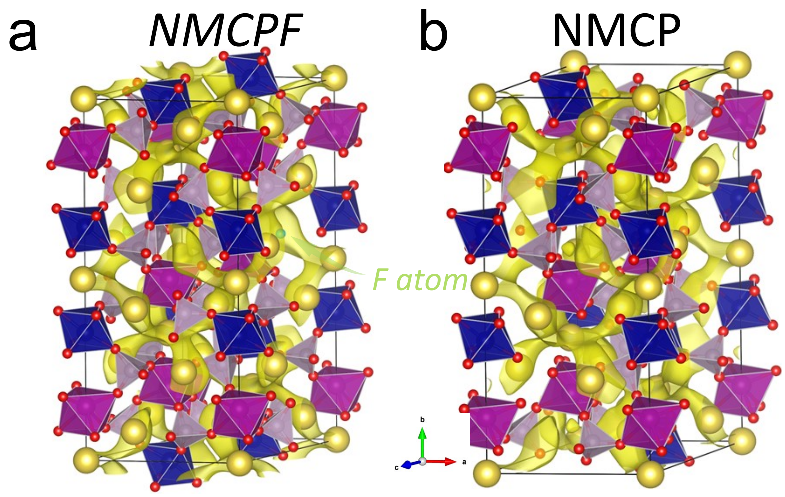

Fig. S1 BV maps of (a) NMCPF and (b) NMCP (the isosurface value is 0.8).

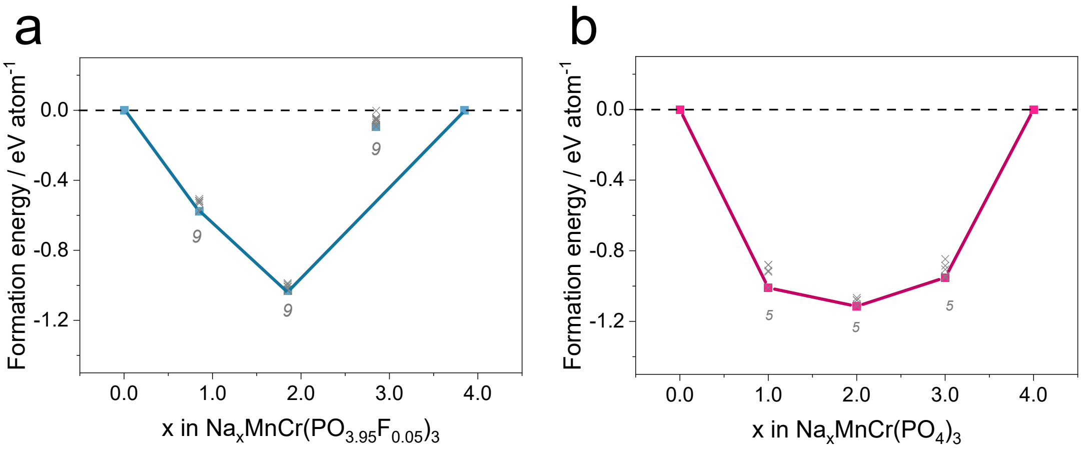

Fig. S2 Convex-hull phase diagrams of (a) NMCPF and (b) NMCP.

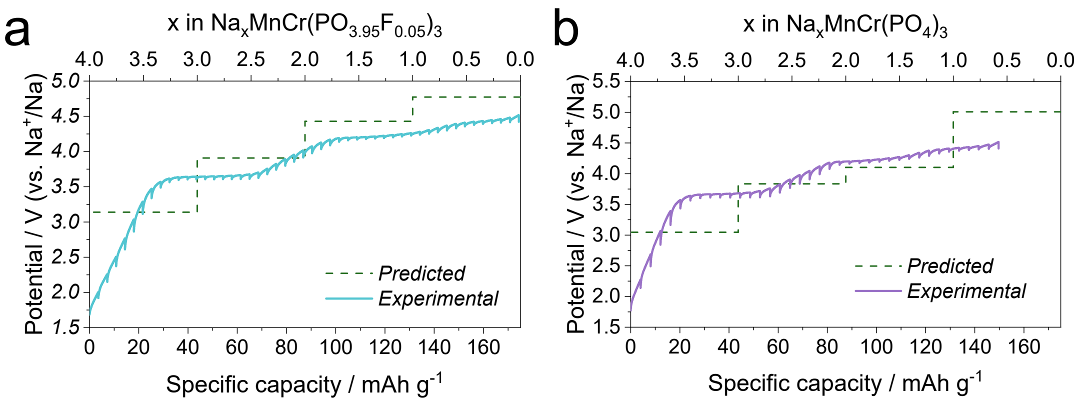

Fig. S3 Calculated voltage profiles of (a) $Na_{3.85}MnCr(PO_{3.95}F_{0.05})_3$ and (b) $Na_4MnCr(PO_4)_3$.

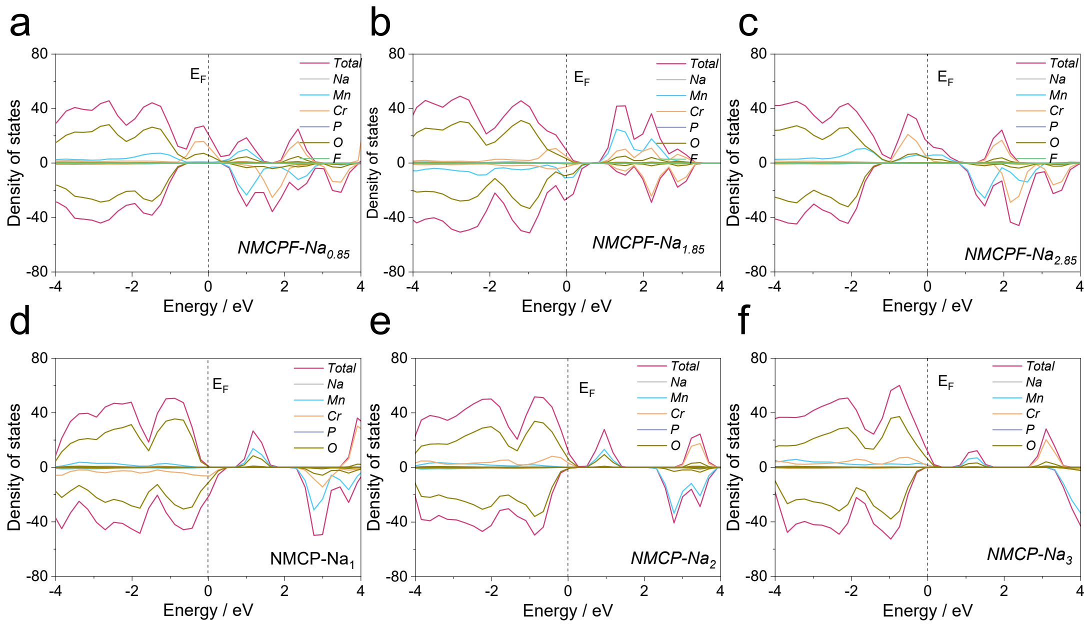

Fig. S4 DOS patterns of (a-c) NMCPF and (d-f) NMCP with different Na contents.

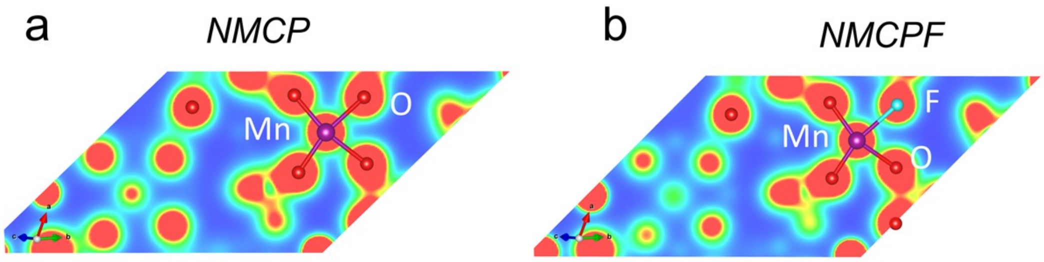

Fig. S5 Illustrations of charge densities of (a) NMCP and (b) NMCPF.

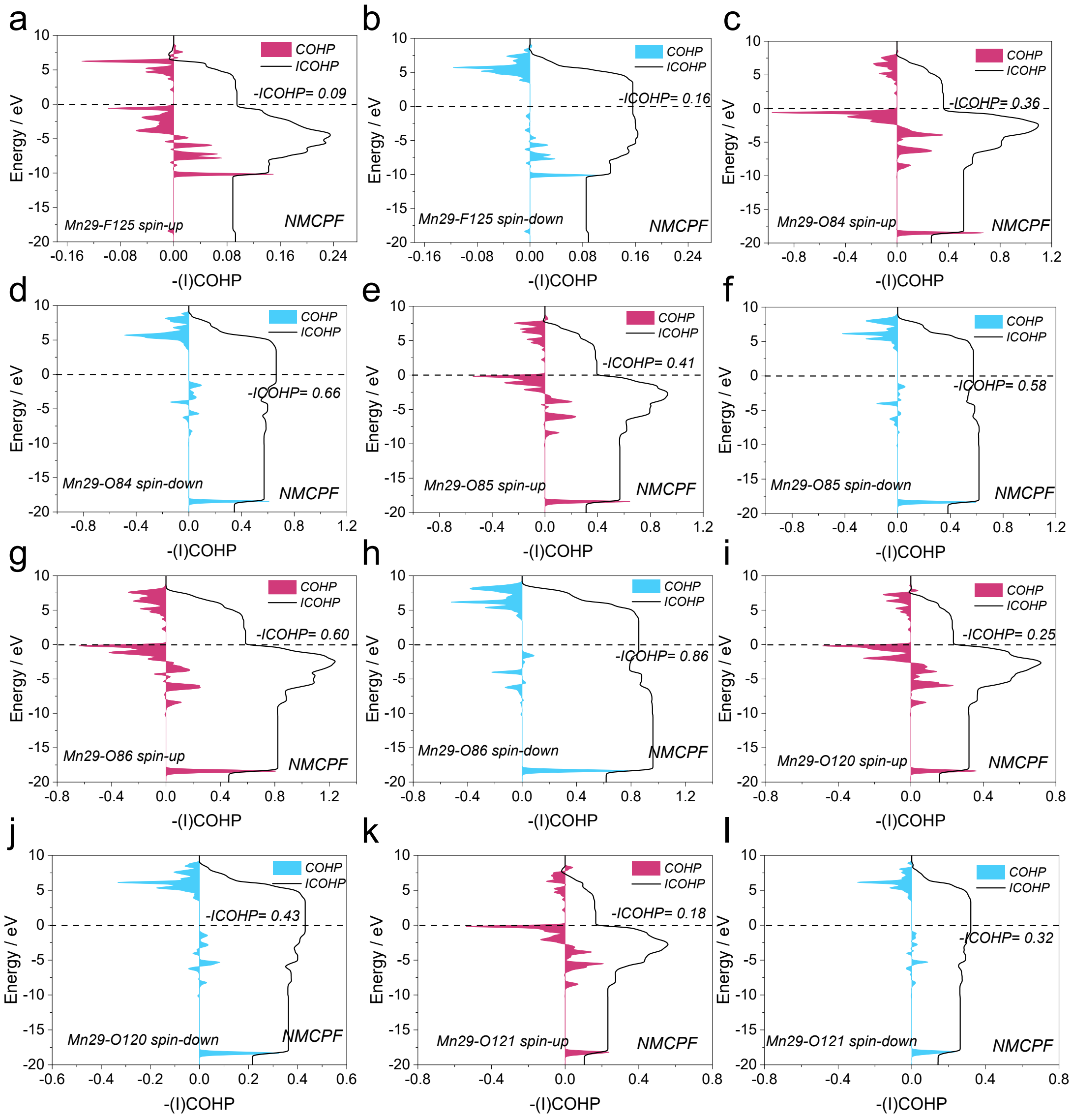

Fig. S6 COHP and ICOHP results of Mn29 atom interacting with different O/F atoms around it in NMCPF.

Table S1. Integrated crystal orbital Hamilton population (ICOHP) of Mn-O or Mn-F bonds in NMCPF.

Type of bond	Distance / Å	Spin direction	ICOHP / eV
Mn29-F125	2.49	Spin up	-0.09
		Spin down	-0.16
Mn29-O121	2.24	Spin up	-0.18
		Spin down	-0.32
Mn29-O120	2.20	Spin up	-0.25
		Spin down	-0.43
Mn29-O86	2.06	Spin up	-0.60
		Spin down	-0.86
Mn29-O85	2.13	Spin up	-0.41
		Spin down	-0.58
Mn29-O84	2.07	Spin up	-0.36
		Spin down	-0.66
	**Integral ICOHP / eV**		-4.91

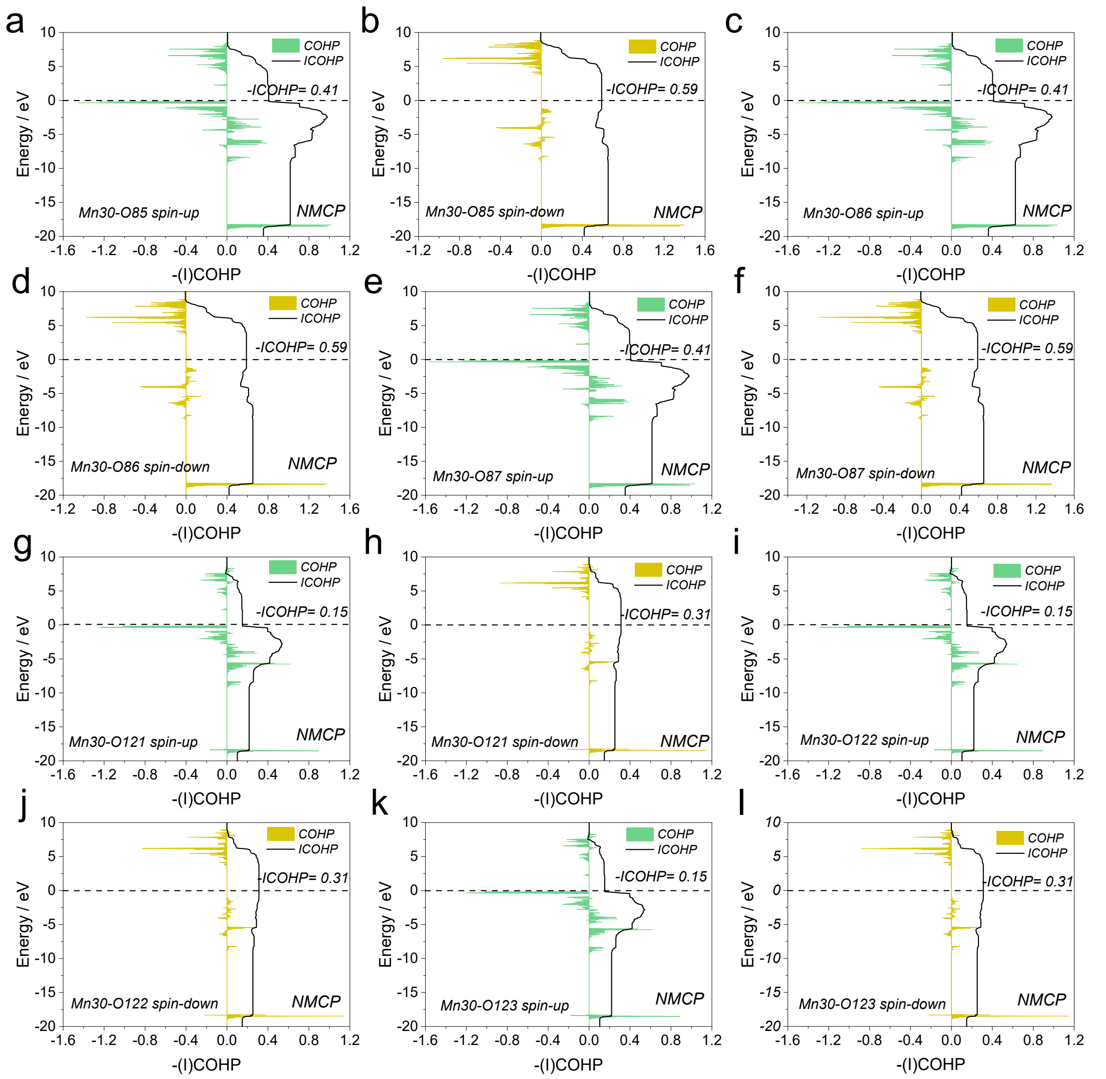

Fig. S7 COHP and ICOHP results of Mn30 atom interacting with different O atoms around it in NMCP.

Table S2. Integrated crystal orbital Hamilton population (ICOHP) of Mn-O bonds in NMCP.

Type of bond	Distance / Å	Spin direction	ICOHP / eV
Mn30-O123	2.25	Spin up	-0.15
		Spin down	-0.31
Mn30-O122	2.25	Spin up	-0.15
		Spin down	-0.31
Mn30-O121	2.25	Spin up	-0.15
		Spin down	-0.31
Mn30-O87	2.12	Spin up	-0.41
		Spin down	-0.59
Mn30-O86	2.12	Spin up	-0.41
		Spin down	-0.59
Mn30-O85	2.12	Spin up	-0.41
		Spin down	-0.59
	**Integral ICOHP / eV**		-4.38

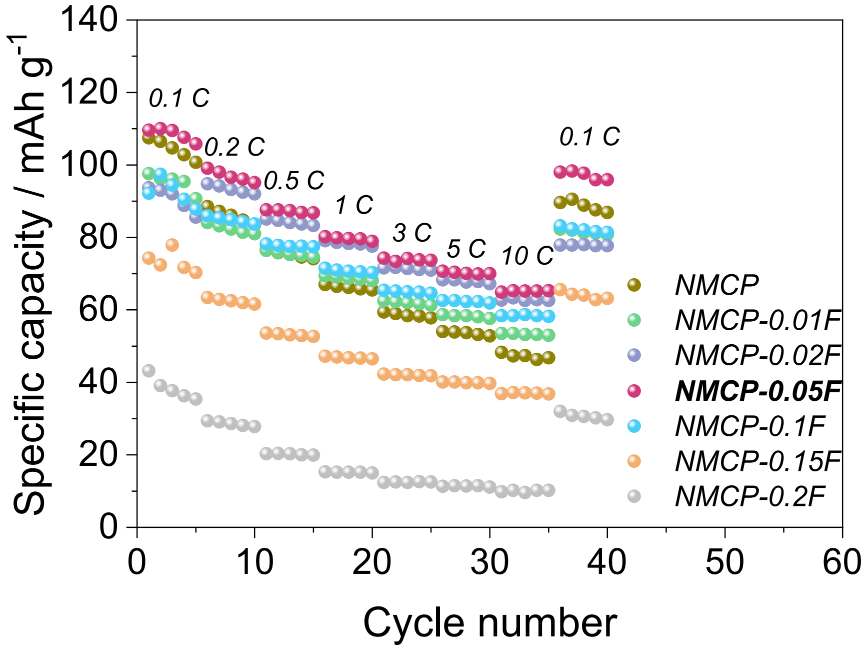

Fig. S8 Rate performances of $Na_{4-3x}MnCr(PO_{4-x}F_x)_3$ samples under 1.5-4.3 V (vs. Na^+/Na).

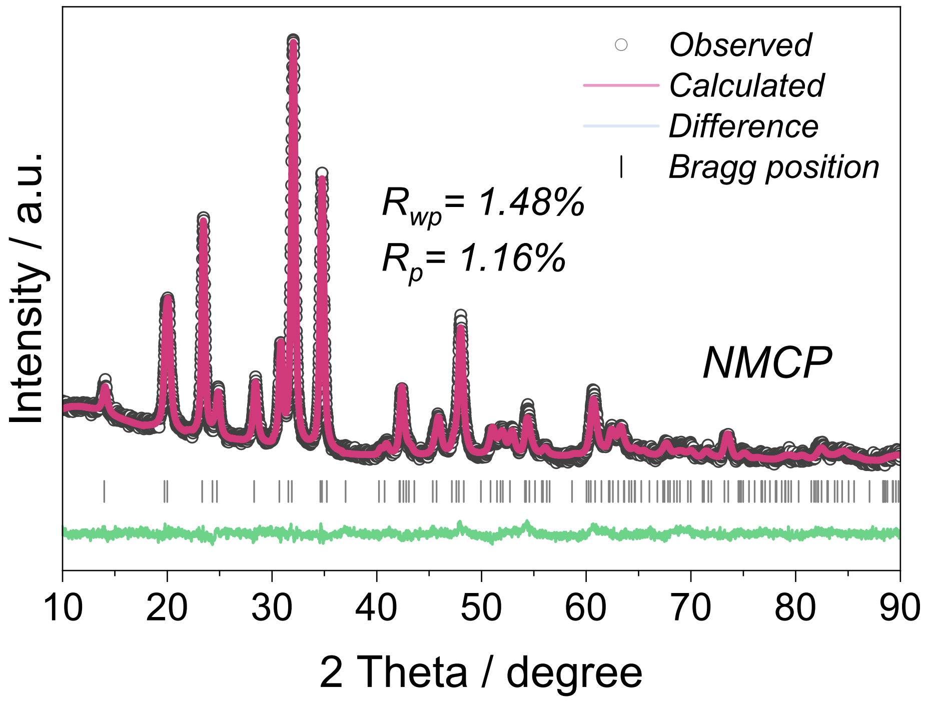

Fig. S9 XRD Rietveld refinement result of NMCP.

Table S3. Detailed structural information of NMCP derived from Rietveld refinement.

Space group = *Rc*		R_{wp} = 1.48%		R_p = 1.16%	
a (Å) = 8.915		c (Å) = 21.422		α (°) = 90	
β (°) = 90		γ (°) = 120		V (Å^3) = 1474.323	
Atom	x	y	z	frac	site
Na1	0.00000	0.00000	0.00000	1.040	6b
Na2	0.64984	0.00000	0.25000	0.971	18e
Mn	0.00000	0.00000	0.14935	0.500	12c
Cr	0.00000	0.00000	0.14935	0.500	12c
P	0.29936	0.00000	0.25000	1.000	18e
O1	0.01594	0.21084	0.19159	1.000	36f
O2	0.18785	0.17284	0.08423	1.000	36f

Table S4. Detailed structural information of NMCPF derived from Rietveld refinement.

Space group = *Rc*		R_{wp} = 1.52%		R_p = 1.18%	
a (Å) = 8.907		c (Å) = 21.409		α (°) = 90	
β (°) = 90		γ (°) = 120		V ($Å^3$) = 1470.906	
Atom	x	y	z	frac	site
Na1	0.00000	0.00000	0.00000	1.058	6b
Na2	0.63891	0.00000	0.25000	0.948	18e
Mn	0.00000	0.00000	0.14920	0.500	12c
Cr	0.00000	0.00000	0.14920	0.500	12c
P	0.29689	0.00000	0.25000	1.000	18e
O1	0.02285	0.20794	0.19536	0.988	36f
O2	0.18276	0.17623	0.08464	0.988	36f
F1	0.02285	0.20794	0.19536	0.012	36f
F2	0.18276	0.17623	0.08464	0.012	36f

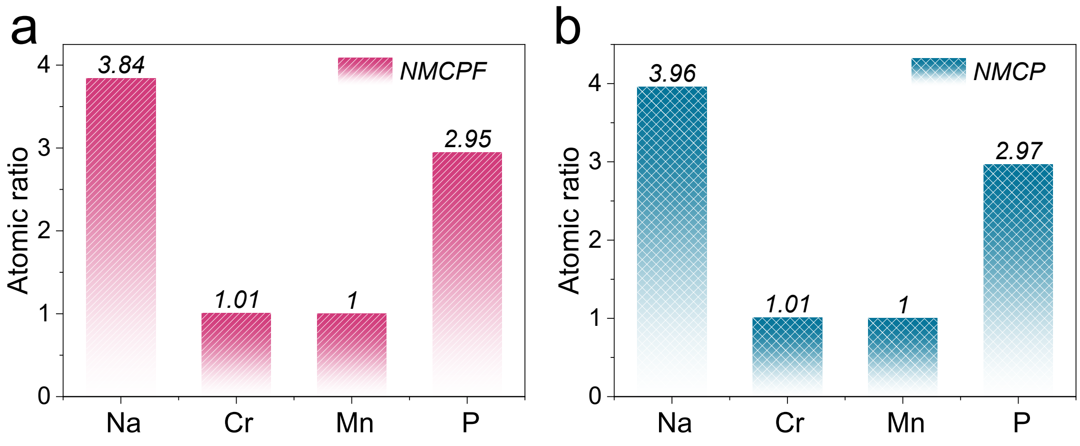

Fig. S10 ICP-OES results of (a) NMCPF and (b) NMCP.

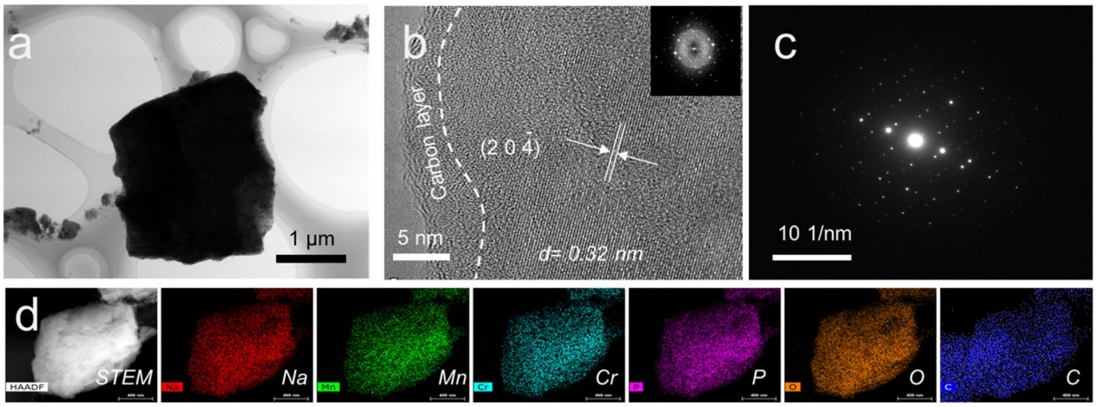

Fig. S11 (a) TEM and (b) HRTEM images (inset: FFT pattern), (c) SAED pattern and (d) EDS mapping images of NMCP.

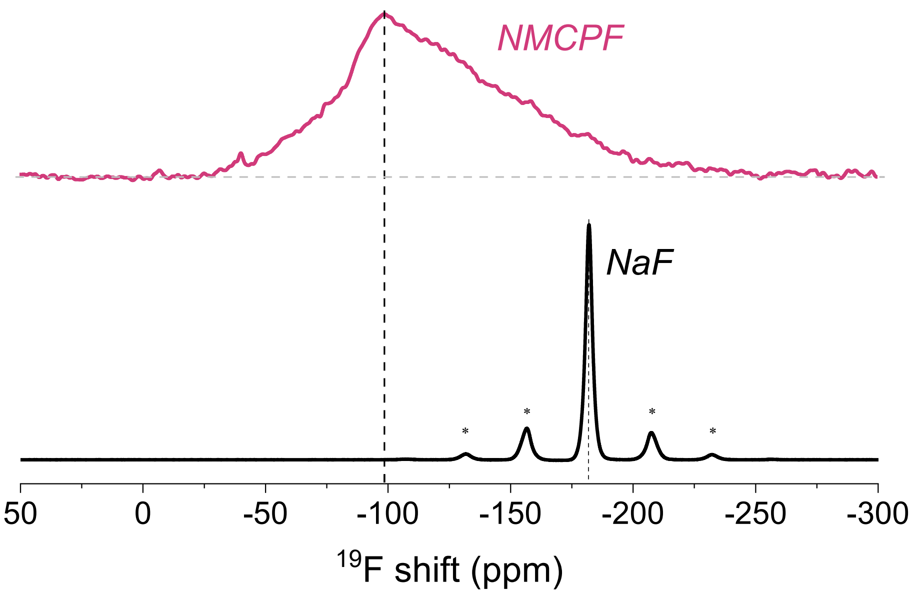

Fig. S12 Solid-state ^{19}F NMR spectra of the pristine NMCPF and the reference NaF. The asterisks indicate spinning sidebands (SSBs).

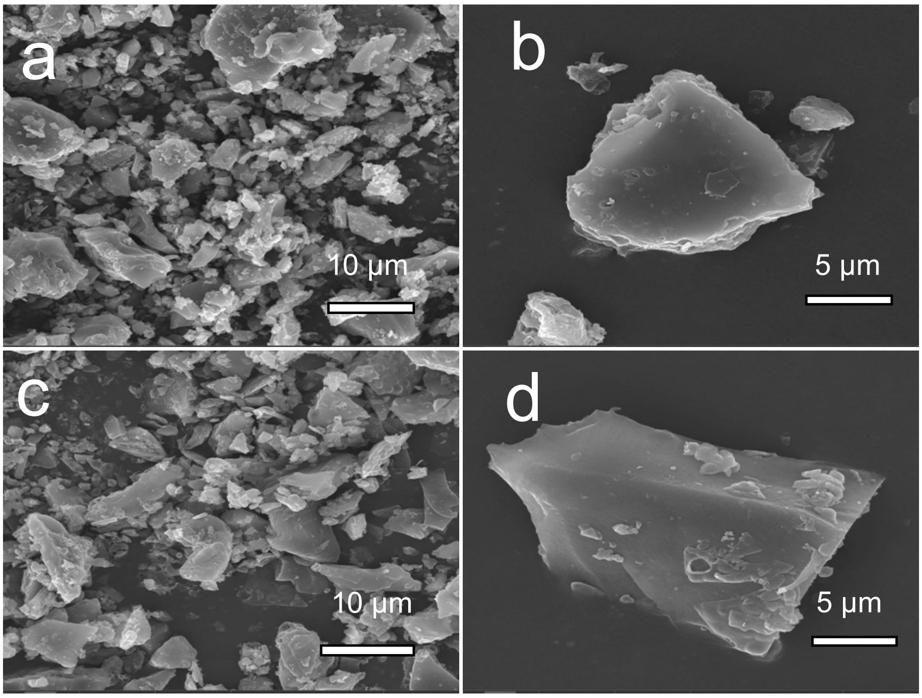

Fig. S13 SEM images of (a-b) NMCPF and (c-d) NMCP.

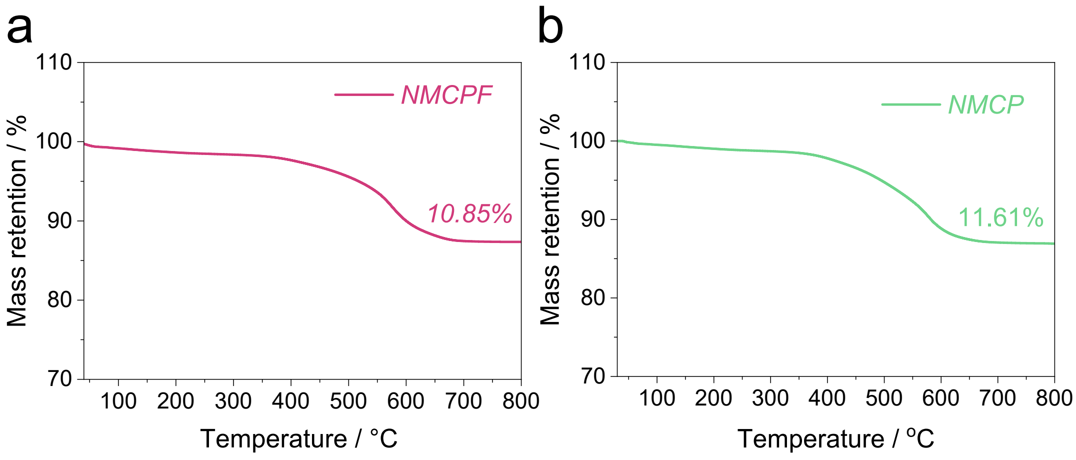

Fig. S14 TGA results of (a) NMCPF and (b) NMCP.

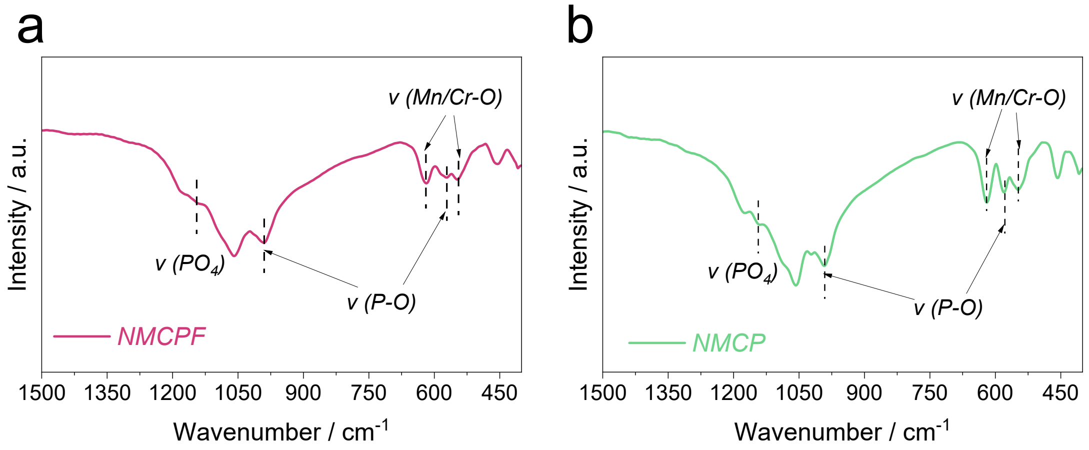

Fig. S15 FT-IR results of (a) NMCPF and (b) NMCP.

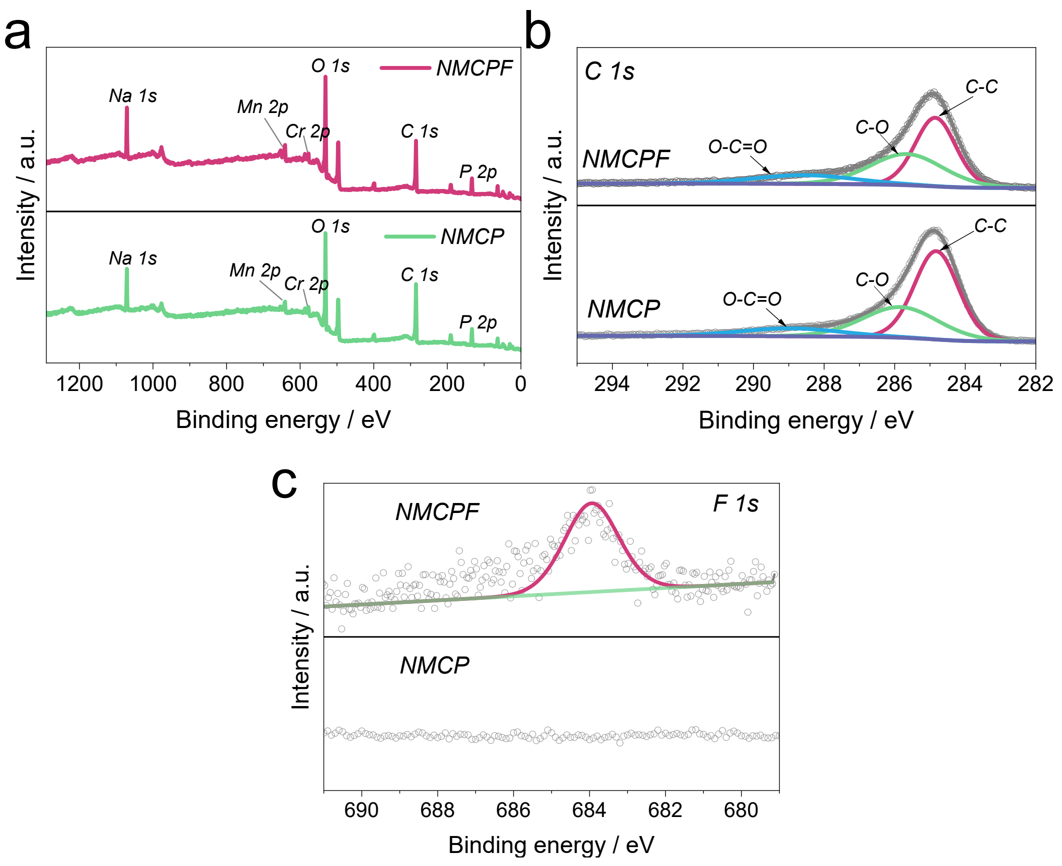

Fig. S16 (a) XPS survey spectra, (b) C 1s and (c) F 1s spectra.

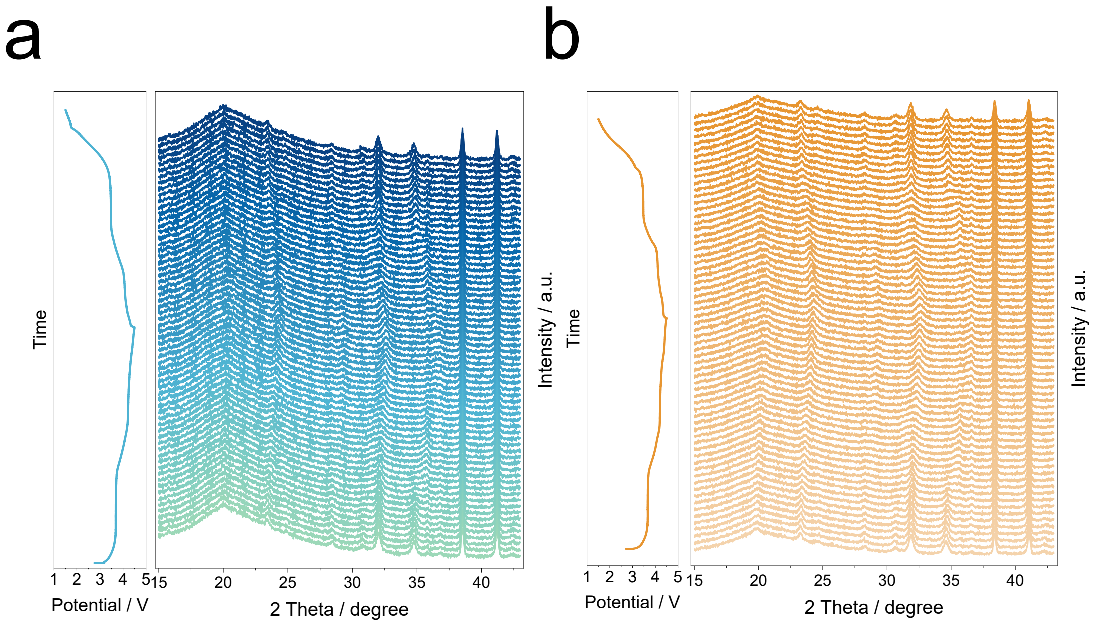

Fig. S17 Full scope of *in-situ* XRD patterns of (a) NMCPF and (b) NMCP.

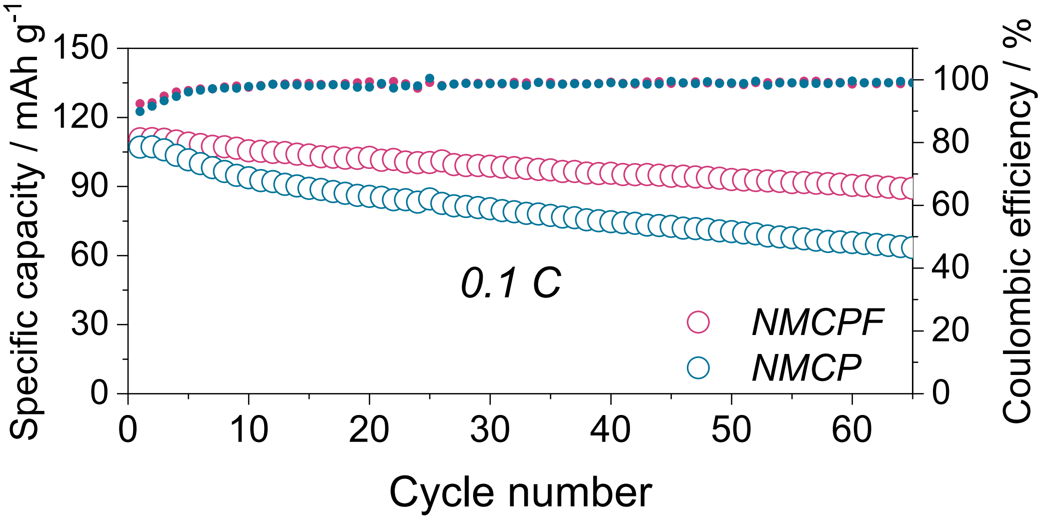

Fig. S18 Cycling stability at 0.1 C within 1.5–4.3 V.

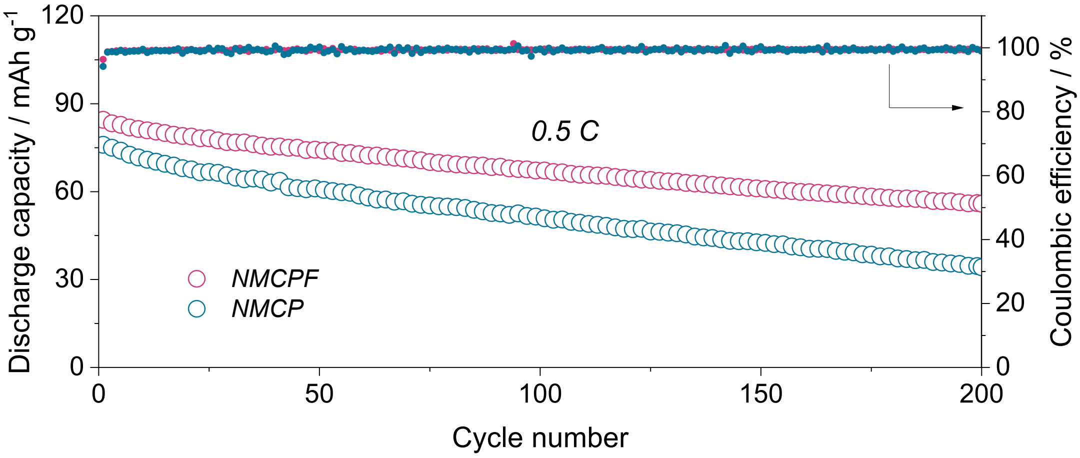

Fig. S19 Cycling stability at 0.5 C within 1.5–4.3 V.

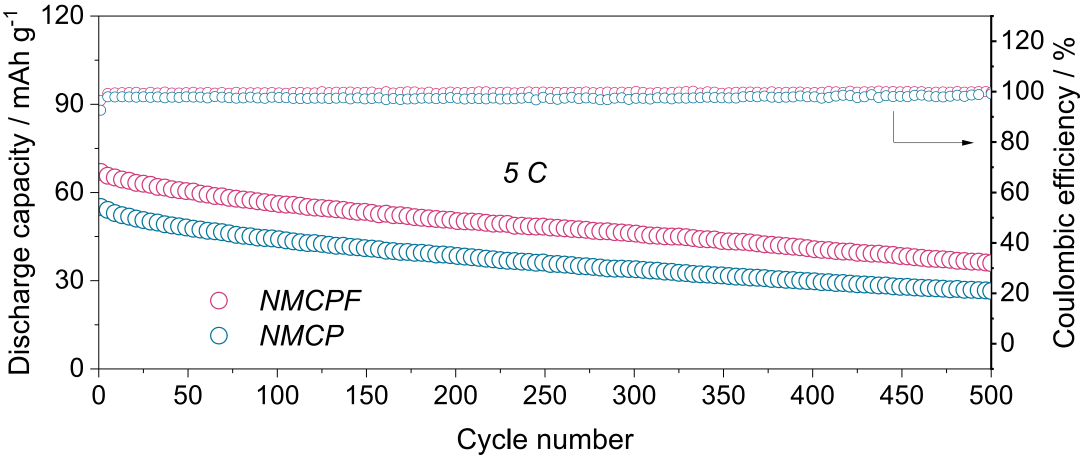

Fig. S20 Cycling stability at 5 C within 1.5–4.3 V.

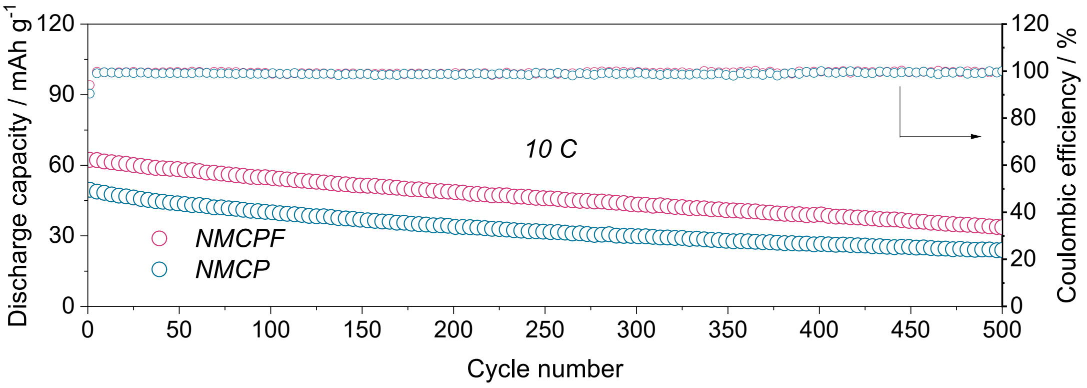

Fig. S21 Cycling stability at 10 C within 1.5–4.3 V.

Table S5. Comparison of cycling stability with other materials proposed in literature.

Materials	Voltage range (V *vs.* Na^+/Na)	Cycling stability	Ref.
NMCPF	1.5-4.3	Capacity retention of 55.1% after 1000 cycles at 20 C	This work
NMCP	2.5-4.6	Capacity retention of 73.3% after 500 cycles at 100 mA g^{-1}	[13]
$Na_2VTi(PO_4)_3$@C	1.5-4.5	Capacity retention of 77% after 500 cycles at 10 C	[14]
$Na_3V_{1.3}Cr_{0.7}(PO_4)_3$	2.5-4.4	Capacity retention of 77.6% after 250 cycles at 0.5 A g^{-1}	[15]
$Na_3MnZr(PO_4)_3$	2.5-4.3	Capacity retention of 91% after 500 cycles at 0.5 C	[16]
$Na_3MnTi(PO_4)_3$	2.5-4.2	Capacity retention of 77.5% after 100 cycles at 0.1 C	[17]
$Na_{3.2}MnTi_{0.8}V_{0.2}(PO_4)_3$	1.5-4.4	Capacity retention of ~68% after 800 cycles at 1 A g^{-1}	[18]
$Na_2MnP_2O_7$@graphene	1.5-4.5	Capacity retention of 60.2% after 1200 cycles at 5 C	[19]

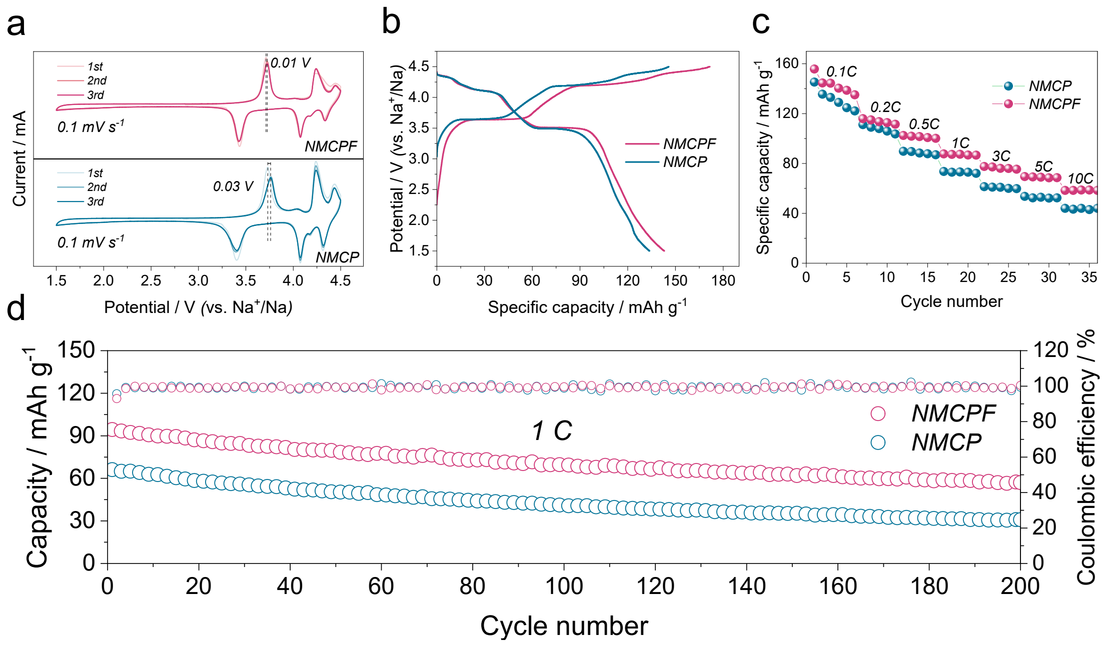

Fig. S22 Electrochemical capabilities within 1.5–4.5 V. (a) CV curves at 0.1 mV s^{-1}. (b) Initial charge/discharge curves at 0.1 C. (c) Rate properties. (d) Cycling capabilities at 1 C.

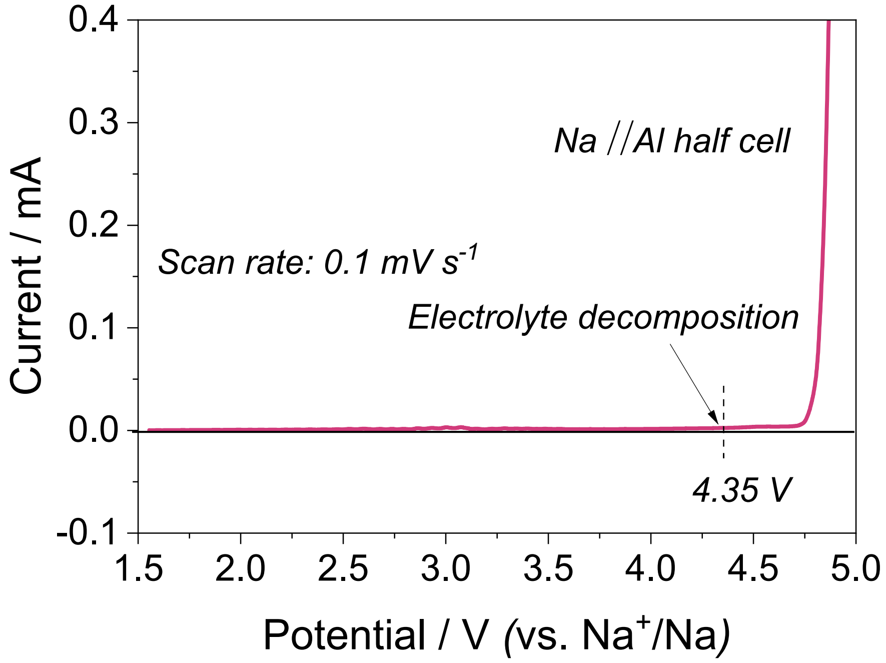

Fig. S23 Linear sweep voltammetry (LSV) curve of the electrolyte (1.0 M $NaClO_4$ in PC and EC solvents with 5 vol.% FEC) at a scan rate of 0.1 mV s^{-1} used in this work.

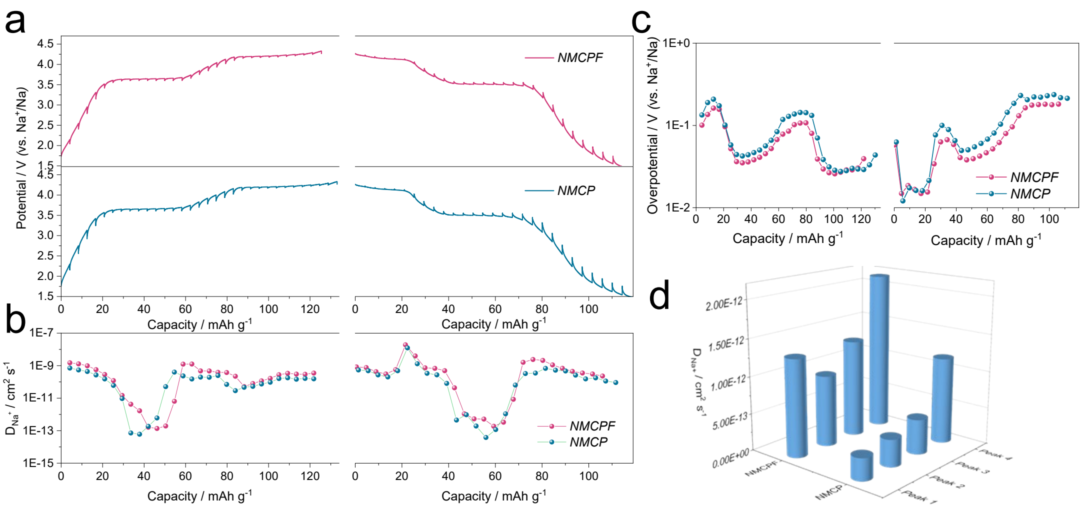

Fig. S24 Kinetics properties of NMCPF and NMCP. (a) GITT profiles and corresponding (b) D_{Na+} as well as (c) overpotentials. (d) D_{Na+} obtained from CV methods at various scan rates.

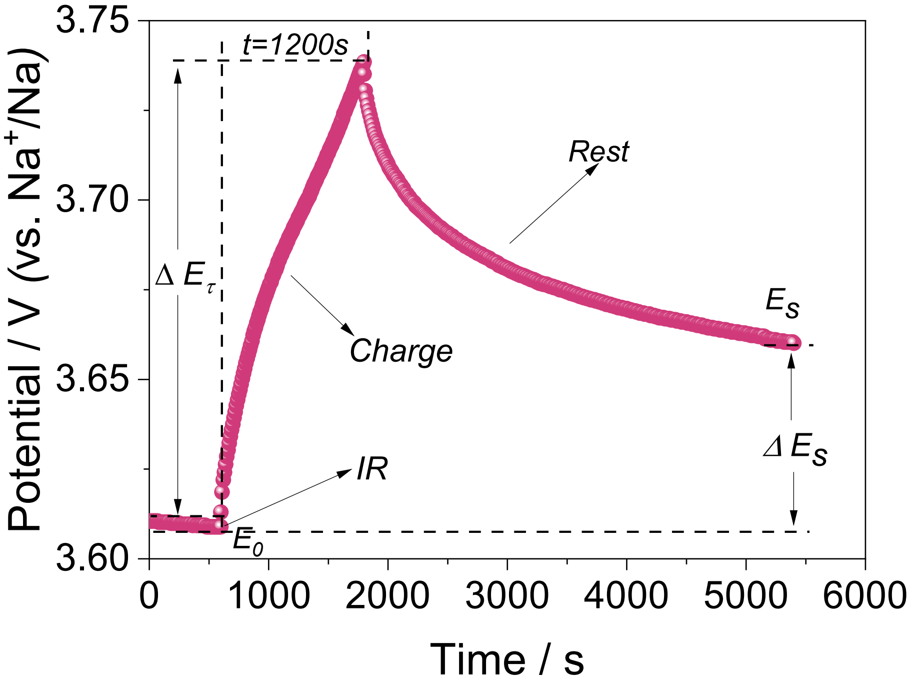

Fig. S25 Detailed schematic diagram of a single-step GITT experiment at ~3.6 V for NMCPF cathode (current density: 0.1 C, 11 mA g^{-1}).

GITT calculation. 1200 s (τ) was set as the charging time, followed by a rest time of 3600 s. To calculate the diffusion coefficient of Na^+ (*D*), the equation is as follow:[20]

$$D = \frac{4}{\pi\tau}\left(\frac{m_B V_m}{M_B S}\right)^2\left(\frac{\Delta E_s}{\Delta E_\tau}\right)^2 \qquad \left(\tau \ll L^2/D\right) \tag{Equation S1}$$

Where L, m_B, M_B, V_m, S represent the thickness of the electrode, mass, molecular weight, molar volume, surface area of NMCPF or NMCP.

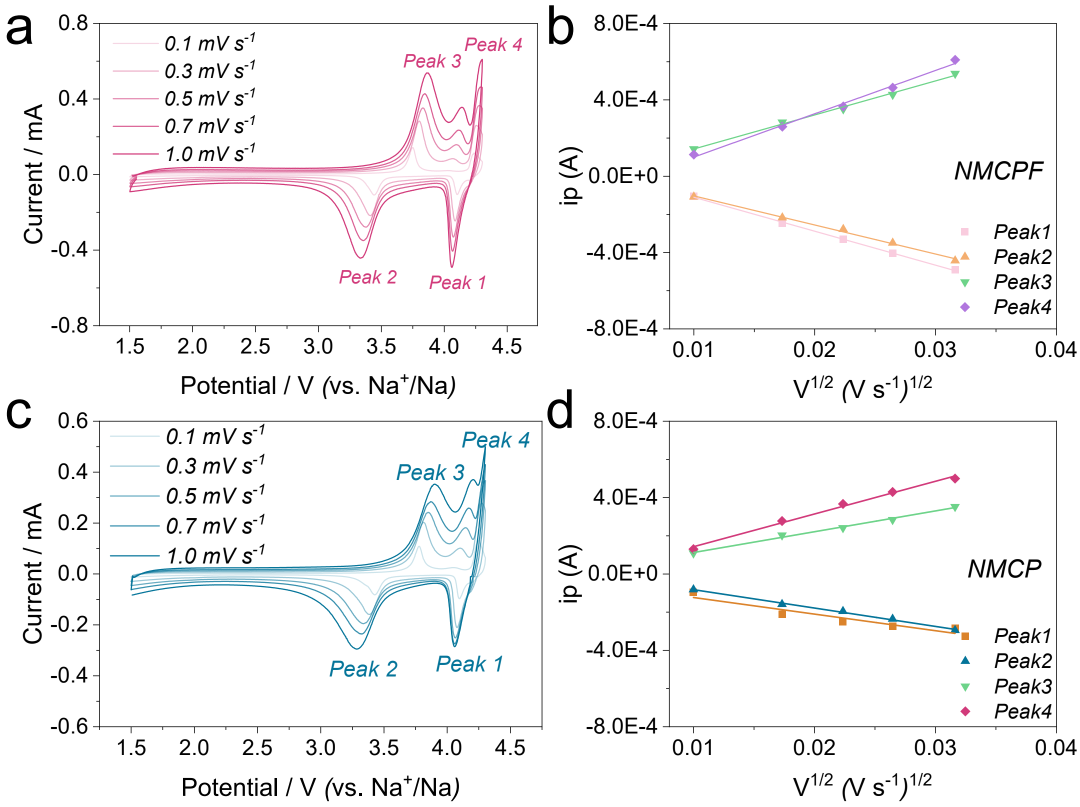

Fig. S26 (a) CV curves at various scan rates from 0.1 to 1.0 mV s^{-1} and (b) the linear fitting for the relationship between i_p and $v^{1/2}$ from CV profiles of NMCPF. (c) CV curves at various scan rates from 0.1 to 1.0 mV s^{-1} and (d) the linear fitting for the relationship between i_p and $v^{1/2}$ from CV profiles of NMCP.

The D_{Na+} from CV tests can be calculated based on the Randles-Sevcik equation:[10-11]

$$i_p = 2.69 \times 10^5 n^{3/2} A D^{1/2} C v^{1/2} \quad \text{(Equation S2)}$$

Where i_p refers to the peak current, n (2 in this test) is the amounts of transferred electrons, A (0.785 cm^2) is the surface area of electrode, C represents the Na^+ molar concentration (2.66×10^{-2} mol cm^{-3}), and v refers to the scan rates.

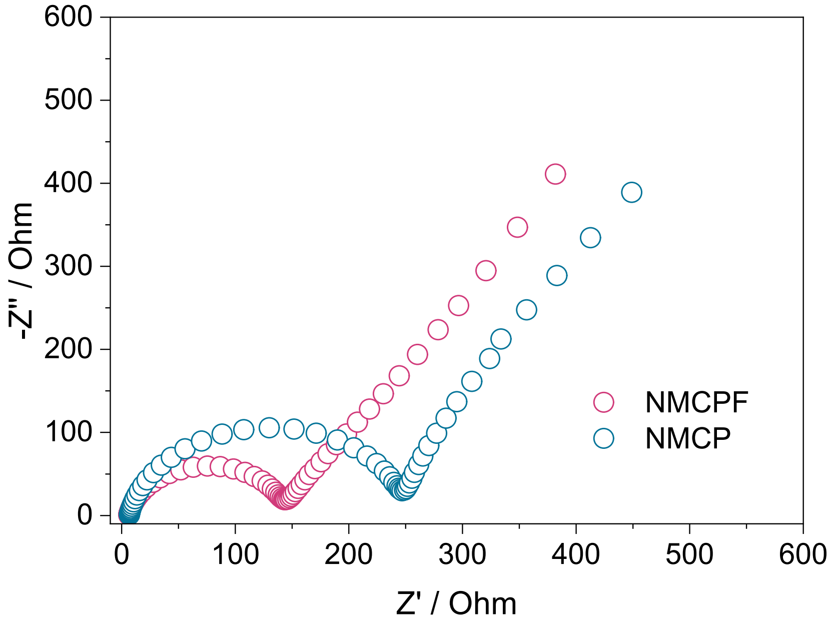

Fig. S27 EIS results of NMCPF and NMCP.

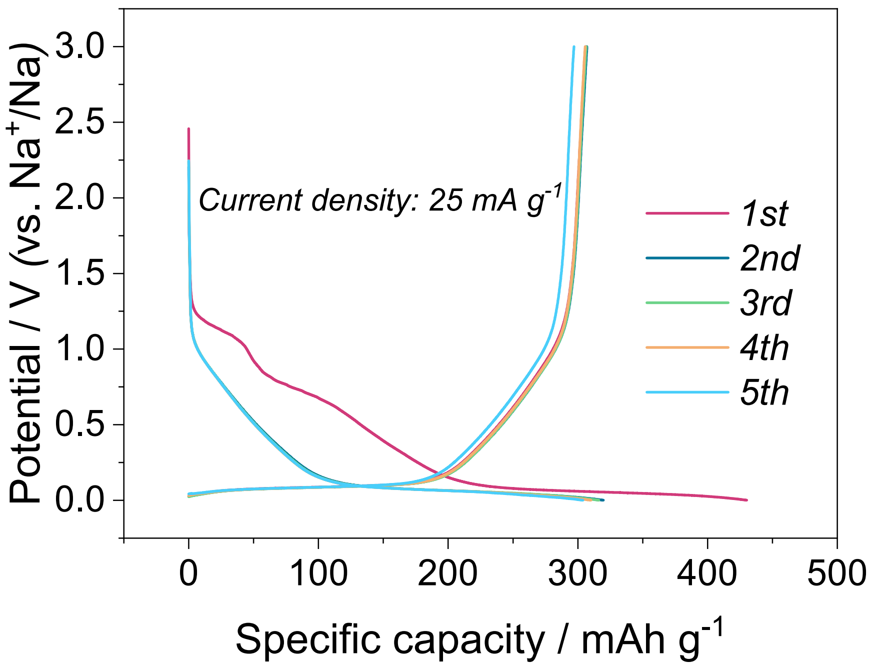

Fig. S28 Galvanostatic charge/discharge curves of the hard carbon anode for initial first 5 cycles.

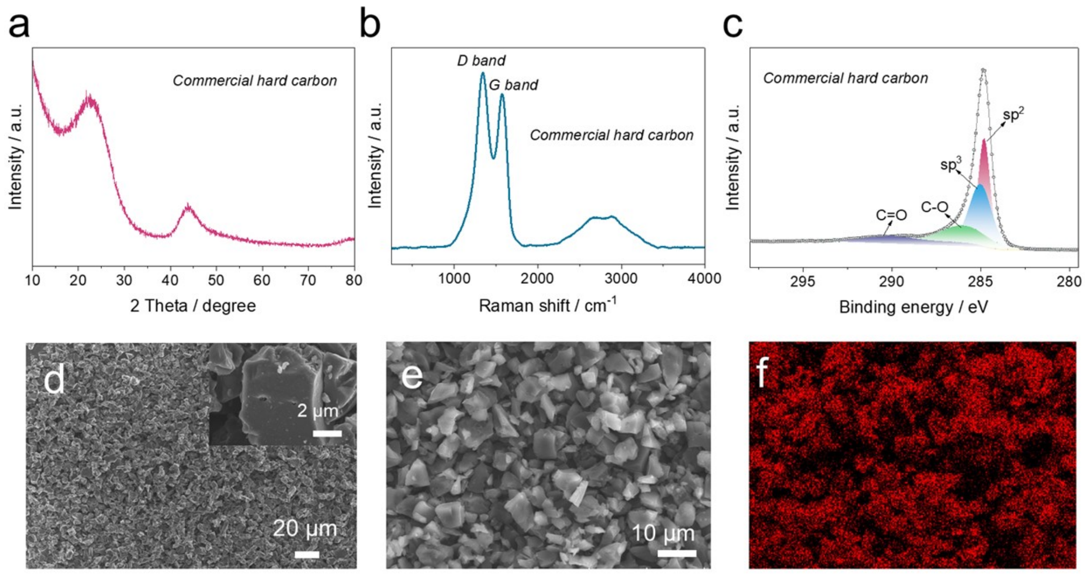

Fig. S29 Characterizations of commercial hard carbon materials. (a) XRD pattern. (b) Raman spectrum. (c) XPS C 1s spectra. (d) SEM images with different resolution. (e-f) EDX mapping images.

We further employed various techniques to characterize the commercial hard carbon material. As seen in Fig. S29a, the XRD pattern presents two broad characteristic peaks located at around 22° and 44°, which can be indexed to (002) and (100) peaks, respectively. The Raman spectrum displays two obvious peaks centered at ~1341.8 cm^{-1} (D band) and ~1575.7 cm^{-1} (G band), which are assigned to the A_{1g} symmetry vibration mode (the sp^2 carbon atoms of disordered graphite) and the E_{2g} symmetry vibration mode of sp^2 carbon atoms in chains and rings, separately. The intensity ratio of D band and G band (I_D/I_G) is measured to be 1.14, indicating it possesses many defects. Besides, Fig. S29c shows four fitted peaks at 284.8 eV, 285.1 eV, 286.2 eV, and 290 eV that can be assigned to be sp^2, sp^3, C-O, and C=O, respectively. Fig. S29d-e present irregular morphologies of hard carbon materials with different particle sizes and relatively smooth surface (inset of Fig. S29d) and uniform elemental distribution.

Supplementary references

1 B. H. Toby, *J. Appl. Crystallogr.*, 2001, **34**, 210-213.

2 S. C. Vogel, *J. Appl. Crystallogr.*, 2011, **44**, 873-877.

3 K. Momma and F. Izumi, *J. Appl. Crystallogr.*, 2011, **44**, 1272-1276.

4 W. Kohn and L. J. Sham, *Phys. Rev.*, 1965, **140**, A1133-A1138.

5 P. Giannozzi, S. Baroni, N. Bonini, M. Calandra, R. Car, C. Cavazzoni, D. Ceresoli, G. L. Chiarotti, M. Cococcioni, I. Dabo, A. Dal Corso, S. de Gironcoli, S. Fabris, G. Fratesi, R. Gebauer, U. Gerstmann, C. Gougoussis, A. Kokalj, M. Lazzeri, L. Martin-Samos, N. Marzari, F. Mauri, R. Mazzarello, S. Paolini, A. Pasquarello, L. Paulatto, C. Sbraccia, S. Scandolo, G. Sclauzero, A. P. Seitsonen, A. Smogunov, P. Umari and R. M. Wentzcovitch, *J. Phys. Condens. Matter.*, 2009, **21**, 395502.

6 H. J. Monkhorst and J. D. Pack, *Phys. Rev. B*, 1976, **13**, 5188-5192.

7 S. L. Dudarev, G. A. Botton, S. Y. Savrasov, C. J. Humphreys and A. P. Sutton, *Phys. Rev. B*, 1998, **57**, 1505-1509.

8 R. Xiao, H. Li and L. Chen, *Chem. Mater.*, 2012, **24**, 4242-4251.

9 G. L. W. Hart and R. W. Forcade, *Phys. Rev. B*, 2008, **77**, 224115.

10 S. P. Ong, W. D. Richards, A. Jain, G. Hautier, M. Kocher, S. Cholia, D. Gunter, V. L. Chevrier, K. A. Persson and G. Ceder, *Comput. Mater. Sci.*, 2013, **68**, 314-319.

11 S. Maintz, V. L. Deringer, A. L. Tchougreeff and R. Dronskowski, *J. Comput. Chem.*, 2016, **37**, 1030-1035.

12 S. P. Ong, V. L. Chevrier, G. Hautier, A. Jain, C. Moore, S. Kim, X. Ma and G. Ceder, *Energy Environ. Sci.*, 2011, **4**, 3680-3688.

13 Y. Zhao, X. Gao, H. Gao, A. Dolocan and J. B. Goodenough, *Nano Lett.*, 2021, **21**, 2281–2287.

14 D. Wang, X. Bie, Q. Fu, D. Dixon, N. Bramnik, Y. S. Hu, F. Fauth, Y. Wei, H. Ehrenberg, G. Chen and F. Du, *Nat. Commun.*, 2017, **8**, 15888.

15 Q. Wang, Y. Zhao, J. Gao, H. Geng, J. Li and H. Jin, *ACS Appl. Mater. Interfaces*, 2020, **12**, 50315-50323.

16 H. Gao, I. D. Seymour, S. Xin, L. Xue, G. Henkelman and J. B. Goodenough, *J. Am. Chem. Soc.*, 2018, **140**, 18192-18199.

17 H. Gao, Y. Li, K. Park and J. B. Goodenough, *Chem. Mater.*, 2016, **28**, 6553-6559.

18 P. Hu, T. Zhu, C. Cai, X. Wang, L. Zhang, L. Mai and L. Zhou, *Angew. Chem. Int. Ed.*, 2023, **62**, e202219304.

19 H. Li, X. Chen, T. Jin, W. Bao, Z. Zhang and L. Jiao, *Energy Storage Mater.*, 2019, **16**, 383-390.

20 X. H. Rui, N. Ding, J. Liu, C. Li and C. H. Chen, *Electrochim. Acta*, 2010, **55**, 2384-2390.
